# Underestimated associated features in CMT neuropathies: clinical indicators for the causative gene?

**DOI:** 10.1002/brb3.451

**Published:** 2016-03-04

**Authors:** Friederike Werheid, Hamid Azzedine, Eva Zwerenz, Ahmet Bozkurt, Marcus J. Moeller, Lilian Lin, Michael Mull, Martin Häusler, Jörg B. Schulz, Joachim Weis, Kristl G. Claeys

**Affiliations:** ^1^ Department of NeurologyUniversity Hospital RWTH AachenAachenGermany; ^2^ Institute of NeuropathologyUniversity Hospital RWTH AachenAachenGermany; ^3^ Department of Plastic and Reconstructive SurgeryHand Surgery‐Burn CenterUniversity Hospital RWTH AachenAachenGermany; ^4^ Department of Plastic & Aesthetic, Reconstructive & Hand SurgeryCenter for Reconstructive Microsurgery and Peripheral Nerve Surgery (ZEMPEN)Agaplesion Markus HospitalFrankfurt am MainGermany; ^5^ Section Immunology and NephrologyDepartment of Internal MedicineUniversity Hospital RWTH AachenAachenGermany; ^6^ Department of NeuroradiologyUniversity Hospital RWTH AachenAachenGermany; ^7^ Division of Neuropediatrics and Social PediatricsDepartment of PediatricsUniversity Hospital RWTH AachenAachenGermany; ^8^ JARA – Translational Brain MedicineAachenGermany; ^9^ Department of NeurologyUniversity Hospitals Leuven and University of Leuven (KU Leuven)LeuvenBelgium

**Keywords:** Additional symptoms, hereditary motor and sensory neuropathy, HMSN, pupillary abnormalities, RLS

## Abstract

**Introduction:**

Charcot–Marie–Tooth neuropathy (CMT) is a genetically heterogeneous group of peripheral neuropathies. In addition to the classical clinical phenotype, additional features can occur.

**Methods:**

We studied a wide range of additional features in a cohort of 49 genetically confirmed CMT patients and performed a systematic literature revision.

**Results:**

Patients harbored a *PMP22* gene alteration (*n* = 28) or a mutation in *MPZ* (*n* = 11), *GJB1* (*n* = 4), *LITAF* (*n* = 2), *MFN2* (*n* = 2), *INF2* (*n* = 1), *NEFL* (*n* = 1). We identified four novel mutations (3 *MPZ,* 1 *GJB1*). A total of 88% presented at least one additional feature. In *MPZ* patients, we detected hypertrophic nerve roots in 3/4 cases that underwent spinal MRI, and pupillary abnormalities in 27%. In our cohort, restless legs syndrome (RLS) was present in 18%. We describe for the first time RLS associated with *LITAF* or *MFN2* and predominant upper limb involvement with *LITAF*. Cold‐induced hand cramps occurred in 10% (*PMP22*,*MPZ*,*MFN2*), and autonomous nervous system involvement in 18% (*PMP22*,*MPZ, LITAF*,*MFN2*). RLS and respiratory insufficiency were mostly associated with severe neuropathy, and pupillary abnormalities with mild to moderate neuropathy.

**Conclusions:**

In CMT patients, additional features occur frequently. Some of them might be helpful in orienting genetic diagnosis. Our data broaden the clinical spectrum and genotype–phenotype associations with CMT.

## Introduction

Charcot–Marie–Tooth (CMT) neuropathy (also called hereditary motor and sensory neuropathy or HMSN) is a rare disorder with a prevalence of one in 2500 (Skre 1974). CMT is clinically characterized by muscle wasting and weakness in the distal limbs resulting in steppage gait as well as distal sensory loss. Foot deformities such as pes cavus and hammer toes are frequently present (Rossor et al. 2013; Timmerman et al. 2014). Usually, the disease course is slowly progressive and in some cases, the use of a walker or wheelchair is necessary later in life (Timmerman et al. 2014). Besides the classical clinical appearance of CMT patients, additional symptoms have been described in association with mutations in certain genes, such as pupil abnormalities with mutations in the myelin protein zero (*MPZ*) gene (De Jonghe et al. 1999; Hattori et al. 2003; Stojkovic et al. 2003), hearing impairment in association with pathogenic variants in the gap junction protein beta 1 (*GJB1*) gene (Hattori et al. 2003), or focal segmental glomerular sclerosis (FSGS) with mutations in the inverted formin 2 (*INF2*) gene (Boyer et al. 2011; Mademan et al. 2013).

CMT has been subdivided into three forms by nerve conduction velocity (NCV) studies: a demyelinating form with motor NCV in the upper limbs below 38 m/sec (CMT1), an axonal form with NCV above 38 m/sec (CMT2) (Harding and Thomas 1980), and an intermediate form with NCV between 25 and 45 m/sec (Davis et al. 1978). The neuropathological hallmark of CMT1 is segmental de‐ and remyelination and onion bulb formations. In CMT2 patients, nerve biopsies typically show axonal loss and regenerative sprouting (Schröder 2001). Both histopathological abnormalities are present in intermediate CMT. At the molecular genetic level, CMT is very heterogeneous with mutations in over 80 causative genes known so far and all possible modes of inheritance (Azzedine et al. 2012; Timmerman et al. 2014). The most frequently mutated gene is peripheral myelin protein 22 (*PMP22*), leading to CMT1A (duplication or point mutation) or hereditary neuropathy with liability to pressure palsies (HNPP, deletion or point mutation) (van Paassen et al. 2014). Novel parallel gene sequencing techniques [next‐generation sequencing (NGS), whole‐exome sequencing (WES)] are nowadays available (Rossor et al. 2013); however, the costs for WES in a diagnostic setting are still high and not (yet) covered by health insurances in many countries. Furthermore, the novel genetic techniques usually lead to a large number of variants of which the causative relation with the disease is often unclear.

Here, we studied the occurrence of additional features in a large cohort of genetically defined CMT patients and performed a systematic revision of the literature. We explored whether the presence of diverse associated clinical features, such as hypertrophic nerve roots or pupil abnormalities, might contribute to identify the causative gene in CMT patients. We also examined whether the occurrence of additional features correlated with the CMTNS2 neuropathy severity score.

## Patients and Methods

### Patient selection

We included 49 patients with genetically confirmed CMT or HNPP that were followed in our neuromuscular outpatient clinic (Department of Neurology, University Hospital RWTH Aachen, Germany) from January 2010 to December 2014 (Table 1). In addition, we searched the database of the Department of Nephrology (University Hospital RWTH Aachen, Germany) for patients born after 1970 presenting focal segmental glomerular sclerosis (FSGS) diagnosed on kidney biopsy. We identified 20 FSGS patients and screened them for an additional polyneuropathy, using a questionnaire on neuropathy symptoms and a clinical neurological examination. The study was approved by the ethical committee of the RWTH University Aachen, Germany.

**Table 1:** Genetic data, CMTNS2 scores and CMT subtype of the patients included in the study (*N* = 49)

Patient (Family)	Gender/AAE	CMTNS2/at AAE	Gene	CMT subtype	Mutation	Protein change	SIFT	PolyPhen‐2	Provean Sift	References
**1 (S)**	**M/27**	***2*** **5**	***MPZ***	**CMT1B**	**c.678delC**	**p.S226fs**	**na**	**na**	**na**	**Novel**
2 (F1)	M/51	16	*MPZ*	CMT1B	c.487G>A	p.G163R	Not tolerated	Probably damaging	Deleterious	Street et al. (2002); Eggers et al. (2004)
3 (F1)	M/78	ND	*MPZ*	CMT1B	c.487G>A	p.G163R	Not tolerated	Probably damaging	Deleterious	Street et al. (2002); Eggers et al. (2004)
4 (F1)	F/46	10	*MPZ*	CMT1B	c.487G>A	p.G163R	Not tolerated	Probably damaging	Deleterious	Street et al. (2002); Eggers et al. (2004)
**5**	**F/30**	**2**	***MPZ***	**CMT2‐I/J**	**c.368G>C**	**p.G123A**	**Not tolerated**	**Probably damaging**	**Deleterious**	**Novel**
6	M/68	14	*MPZ*	CMT1B	c.293G>A	p.R98H	Not tolerated	Probably damaging	Deleterious	Gabreels‐Festen et al. (1996); Watanabe et al. (2002)
**7 (F2)(S)**	**F/44**	**29**	***MPZ***	**CMT1B**	**c.103_104insA**	**p.L35fsX66**	**na**	**na**	**na**	**Novel**
**8 (F2)(S)**	**M/70**	**4**	***MPZ***	**CMT1B**	**c.103_104insA**	**p.L35fsX66**	**na**	**na**	**na**	**Novel**
**9 (F2)(S)**	**M/41**	**0**	***MPZ***	**CMT1B**	**c.103_104insA**	**p.L35fsX66**	**na**	**na**	**na**	**Novel**
10	F/48	27	*MPZ*	CMT2‐I/J	c.293G>A	p.R98H	Not tolerated	Probably damaging	Deleterious	Gabreels‐Festen et al. (1996); Watanabe et al. (2002)
11	M/64	22	*MPZ*	CMT1B	c.670G>T	p.D224Y	Not tolerated	Probably damaging	Deleterious	Fabrizi et al. (2006)
12 (F3)	M/33	6	*LITAF*	CMT1C	c.430G>A	p.V144M	Not tolerated	Probably damaging	Damaging	Gerding et al. (2009)
13 (F3)	F/54	22	*LITAF*	CMT1C	c.430G>A	p.V144M	Not tolerated	Probably damaging	Damaging	Gerding et al. (2009)
14	M/51	21	*GJB1*	CMTX1	c.547C>A	p.R183S	Not tolerated	Probably damaging	Deleterious	Bort et al. (1997)
15	F/38	18	*GJB1*	CMTX1	c.8G>A	p.W3X	na	na	na	Ananth U ([Link]), private data
**16 (F4)**	**F/45**	**15**	***GJB1***	**CMTX1**	**c.303dup**	p.E102Rfs*8	**na**	**na**	**na**	**Novel**
**17 (F4)**	**M/39**	**18**	***GJB1***	**CMTX1**	**c.303dup**	p.E102Rfs*8	**na**	**na**	**na**	**Novel**
18	M/48	na	*PMP22*	HNPP	Deletion	Deletion	na	na	na	Chance et al. (1993)
19	M/37	na	*PMP22*	HNPP	Deletion	Deletion	na	na	na	Chance et al. (1993)
20	F/26	na	*PMP22*	HNPP	Deletion	Deletion	na	na	na	Chance et al. (1993)
21	F27	na	*PMP22*	HNPP	Deletion	Deletion	na	na	na	Chance et al. (1993)
22	F/33	na	*PMP22*	HNPP	Deletion	Deletion	na	na	na	Chance et al. (1993)
23	M/51	na	*PMP22*	HNPP	Deletion	Deletion	na	na	na	Chance et al. (1993)
24 (F5)	F/65	na	*PMP22*	HNPP	Deletion	Deletion	na	na	na	Chance et al. (1993)
25 (F5)	F/40	na	*PMP22*	HNPP	Deletion	Deletion	na	na	na	Chance et al. (1993)
26	F/56	na	*PMP22*	HNPP	Deletion	Deletion	na	na	na	Chance et al. (1993)
27 (F6)	F/49	21	*PMP22*	CMT1A	Duplication	Duplication	na	na	na	Lupski et al. (1991); Raeymaekers et al. (1991)
28 (F6)	F/70	18	*PMP22*	CMT1A	Duplication	Duplication	na	na	na	Lupski, et al. (1991); Raeymaekers et al. (1991)
29	M/23	11	*PMP22*	CMT1A	Duplication	Duplication	na	na	na	Lupski et al. (1991); Raeymaekers et al. (1991)
30	F/26	24	*PMP22*	CMT1A	Duplication	Duplication	na	na	na	Lupski et al. (1991); Raeymaekers et al. (1991)
31	M/30	25	*PMP22*	CMT1A	c.256C>T	p.Q86X	na	na	na	Numakura et al. (2002)
32	M/62	7	*PMP22*	CMT1A	Duplication	Duplication	na	na	na	Lupski et al. (1991); Raeymaekers et al. (1991)
33	M/42	14	*PMP22*	CMT1A	Duplication	Duplication	na	na	na	Lupski et al. (1991); Raeymaekers et al. (1991)
34	M/59	26	*PMP22*	CMT1A	Duplication	Duplication	na	na	na	Lupski et al. (1991); Raeymaekers et al. (1991)
35	F/49	31	*PMP22*	CMT1A	Duplication	Duplication	na	na	na	Lupski et al. (1991); Raeymaekers et al. (1991)
36	F/56	21	*PMP22*	CMT1A	Duplication	Duplication	na	na	na	Lupski et al. (1991); Raeymaekers et al. (1991)
37 (F7)	F/66	24	*PMP22*	CMT1A	Duplication	Duplication	na	na	na	Lupski et al. (1991); Raeymaekers et al. (1991)
38 (F7)	M/45	19	*PMP22*	CMT1A	Duplication	Duplication	na	na	na	Lupski et al. (1991); Raeymaekers et al. (1991)
39	F/51	20	*PMP22*	CMT1A	Duplication	Duplication	na	na	na	Lupski et al. (1991); Raeymaekers et al. (1991)
40	M/63	27	*PMP22*	CMT1A	Duplication	Duplication	na	na	na	Lupski et al. (1991); Raeymaekers et al. (1991)
41	F/46	16	*PMP22*	CMT1A	Duplication	Duplication	na	na	na	Lupski et al. (1991); Raeymaekers et al. (1991)
42	F/34	ND	*PMP22*	CMT1A	Duplication	Duplication	na	na	na	Lupski et al. (1991); Raeymaekers et al. (1991)
43 (F8)	F/8	5	*PMP22*	CMT1A	Duplication	Duplication	na	na	na	Lupski et al. (1991); Raeymaekers et al. (1991)
44 (F8)	F/45	24	*PMP22*	CMT1A	Duplication	Duplication	na	na	na	Lupski et al. (1991); Raeymaekers et al. (1991)
45	F/51	26	*PMP22*	CMT1A	Duplication	Duplication	na	na	na	Lupski et al. (1991); Raeymaekers et al. (1991)
46	M/45	13	*NEFL*	CMT1F	c.995A>C	p.Q332P	Not tolerated	Probably damaging	Deleterious	Mersiyanova et al. (2000)
47	F/12	ND	*INF2*	CMTDIE	c.230T>G	p.L77R	Not tolerated	Probably damaging	Deleterious	Mademan et al. (2013)
48	F/32	ND	*MFN2*	CMT2A2	c.839G>A	p.R280H	Not tolerated	Probably damaging	Deleterious	Zuchner et al. (2004); Chung et al. (2006); Verhoeven et al. (2006)
49	M/42	21	*MFN2*	CMT2A2	c.281G>A	p.R94Q	Not tolerated	Probably damaging	Deleterious	Zuchner et al. (2004); Chung et al. (2006); Verhoeven et al. (2006)

AAE, age at examination; CMTNS2, Charcot–Marie–Tooth neuropathy score version 2 (score ranging from 0 to 36); mild: 0–10; moderate: 11–20; severe neuropathy: >20; CMT, Charcot–Marie–Tooth neuropathy; HNPP, hereditary neuropathy with liability to pressure palsies. In bold, the novel mutations are indicated. SIFT, sorting intolerant from tolerant algorithm; PolyPhen‐2, polymorphism phenotyping version 2; Provean Sift, protein variation effect analyzer; S, segregation analysis was performed in the patient's family. Families are marked with F followed by the family number; na, not applicable; ND, not done; Gene abbreviations: *MPZ* = myelin protein zero; *LITAF* = lipopolysaccharide‐induced tumor necrosis factor‐alpha factor; *GJB1 *=* *gap junction protein, beta‐1; *PMP22 *=* *peripheral myelin protein 22; *NEFL* = neurofilament protein, light polypeptide; *INF2 *=* *inverted formin 2; *MFN2 *=* *mitofusin 2.

### Clinical and paraclinical examinations

In all patients, we performed a detailed history taking, and general clinical and neurological examination with particular attention for additional features (Table 2). The feature cold‐induced hand cramps was asked for anamnestically and the presence of scoliosis was derived from the patients' medical reports in a retrospective manner. We applied the CMT neuropathy score version 2 (CMTNS2) in 37 patients, in order to evaluate the severity of neuropathy: mild (range 0–10), moderate (range 11–20), or severe (>20, maximum 36) (Table 1) (Murphy et al. 2011). We systematically performed motor and sensory nerve conduction velocities (NCV) at the upper limbs in all patients and additionally at the lower limbs in some. Prior to this study, a lumbar puncture was performed in two CMT patients (patients 2 and 6), a magnetic resonance imaging (MRI) of the thoraco‐lumbo‐sacral spine (Philips Intera, 1.5 Tesla, Andover, MA) in patients 2, 4, 6, 7, and 13, a MRI of the cervical spine in patient 12 and a MRI of the brain in patients 1, 2, 5, 6, 12, and 22 for diagnostic purposes. Sural nerve biopsies were obtained previous to the study for diagnostic reasons in patients 1, 2, 6, 23, 24, and 47, after written informed consent. The biopsies were processed following standard procedures (Weis et al. 2012). Semithin sections were stained with toluidine blue and both light and electron microscopic studies were performed.

**Table 2:** Additional symptoms and features identified in our CMT cohort (*N* = 49)

Mutated gene	*MPZ*	*PMP22* (CMT1A)	*PMP22* (HNPP)	*GJB1*	*NEFL*	*INF2*	*LITAF*	*MFN2*
Number of patients	11	19	9	4	1	1	2	2
Tremor	+(P3)	+(P30, 35, 38, 41, 45)	+(P19, 22, 24)	+(P14, 17)	−	−	+(P13)	+(P49)
UL predominant	−	−	−	−	−	−	+(P13)	−
Scoliosis	+(P1, 2, 4)	+(P29,31, 35, 37, 39, 41, 42, 43, 45)	+(P25)	+(P15)	−	−	+(P12^a^)	−
Hand deformities	+(P1, 10)	+(P40)	−	−	−	−	−	−
Skeletal deformities	−	Hip dysplasia bilat. (P33, 42, 45)	−	−	−	Ulnar deviation of hands, hyper−kyphosis (P47)	−	−
Deafness	−	+(P35)	−	−	−	−	−	−
Cognitive impairment	+(P1, 7)	+(P30)	−	−	−	−	+(P13)	−
Bulbar	−	Dysphagia (P35)	−	−	−	−	−	−
Fasciculations	+(P10)	−	+(P19)	−	−	−	−	−
Facial weakness	−	+(P28, 30, 32, 45)	+(P18)	−	−	−	−	−
Pain	+(P1, 7)	+(P31, 34, 35, 36, 39, 40, 41, 45)	+(P22)	+(P14, 15)	−	−	+(P12^a^, 13)	+(P48)
Paresthesia	+(P1, 5, 6, 7)	+(P27, 32, 35, 38, 40, 41)	+(P19, 26)	+(P14, 17)	+(P46)	−	+(P12^a^, 13)	+(P48)
Early onset	+(P1)	−	−	−	−	−	−	−
Eye involvement	Pupillary (P2, 4, 5)	−	−	−	−	−	−	−
CTS	+(P4, 7)	+ (P40, 45)	+(P24)	−	−	−	+(P13)	−
Respiratory insufficiency	Restrictive +(P10)	+(P30), elevated diaphragm (P37)	−	−	−	−	−	−
Autonomous	Bladder urgency (P1), postural hypotension (P7), urinary incon‐tinence (P7), hyperhidrosis (P11)	Incontinence (P36), hyperhidrosis (P38), postural hypotension (P34)	Slowed emptying of stomach (P20)	−	−	−	Postural hypoten‐sion (P13)	Bladder urgency (P49)
Brain MRI	Vascular leucencepha‐lopathia and plump lateral ventricles (P6, MRI at age 65y)	−	−	−	−	−	−	−
Hypertrophic nerve roots	+(P2, 4, 6)	−	−	−	−	−	−	−
RLS	+(P1)	+(P40, 45)	+(P24, 26)	+(P17)	−	−	+(P12^a^, 13)	+(P48)
Cold‐induced hand cramps	+(P7)	+(P39, 40)	+(P24)	−	−	−	−	+(P48)
ESRD, proteinuria, GS	−	−	−	−	−	+(P47)	−	−

CMT1A, Charcot–Marie–Tooth neuropathy type 1A; HNPP, hereditary neuropathy with liability to pressure palsies; P, patient; UL, upper limb; bilat., bilateral; CTS, carpal tunnel syndrome; MRI, magnetic resonance imaging; RLS, restless legs syndrome; ESRD, end‐stage renal disease; GS, glomerular sclerosis.

a Multiple sclerosis as additional disease in this patient. For abbreviations of genes, see text and Table 1.

### Molecular genetic analyses

The molecular genetic tests were performed using genomic DNA from total peripheral blood samples following standard procedures, after obtaining patients' written informed consent. Sanger sequencing or multiplex ligation‐dependent probe amplification (MLPA) combined with microsatellite marker method was performed in 46 CMT patients. A molecular genetic analysis of *INF2* was conducted in two patients with a polyneuropathy and histologically proven FSGS (from the initial cohort of 20 patients with FSGS, see above). NGS‐based panel diagnostics for mutations in 71 CMT‐causing genes was done in three patients (patients 6, 10, and 46; Tables 1 and 2), in whom previous single‐gene analyses had not revealed a causative mutation. In addition, a segregation analysis was performed in two families (patient 1 with healthy mother and healthy sister; patients 7, 8, 9 with healthy mother and affected father – family 2; Table 1). We used the prediction programs SIFT (sorting intolerant from tolerant algorithm), PolyPhen2 (polymorphism phenotyping version 2), and Provean Sift (protein variation effect analyzer) to verify pathogenicity of the identified variants (Table 1).

### Literature search and meta‐analysis

In order to study the additional features reported in CMT patients so far, we conducted a systematic review in PubMed from 1985 to 2014. We included published CMT patients in whom a genetic defect was identified, and excluded patients with other hereditary neuropathies, such as hereditary motor neuropathies (HMN) or hereditary sensory (and autonomic) neuropathies (HSN, HSAN). CMT patients with compound heterozygous mutations in two different genes were also excluded, since no unequivocal genotype–phenotype correlation could be made. These criteria led to 280 publications that have been included in this study (Appendix S1).

## Results

### Patient cohort

Our study cohort included 49 patients with a genetically confirmed diagnosis of CMT or HNPP (Table 1). Nineteen patients belonged to eight different families and 30 patients were isolated cases. Six distinct mutations have been identified in the *MPZ* gene, of which three were novel (patients 1, 5; and patients 7, 8, 9 belonging to family 2) and predicted to be pathogenic by the three prediction programs applied (Table 1). Furthermore, the variants co‐segregated with the disease status in the healthy mother and sister of patient 1, as well as in the healthy mother, affected father (patient 8) and brother (patient 9) of index patient 7. The two patients carrying the same c.293G>A, p.R98H mutation in *MPZ* (patients 6 and 10) were not related. Eighteen patients carried a *PMP22* duplication, nine had a *PMP22* deletion, and one harbored a point mutation in the *PMP22* gene. In four patients, a pathogenic variant in the *GJB1* gene was identified, of which the c.303dup, p.E102Rfs*8 mutation in *GJB1* in the related patients 16 and 17 was novel (brother and sister, family 4, Table 1). Two patients belonging to the same family (mother and son) carried a mutation in the lipopolysaccharide‐induced tumor necrosis factor‐*α* factor (*LITAF*) gene, one in the neurofilament protein light polypeptide (*NEFL*) gene, one in *INF2*, and two in the mitofusin 2 (*MFN2*) gene. The patient with *INF2* mutation has been described previously (Roos et al. 2015). Neuropathy severity, measured using the CMTNS2 score, was mild in seven patients (range 0–10), moderate in 13 (range 11–20), and severe in 17 cases (>20). Genetic data, CMTNS2 scores, and CMT subtype of the patients are summarized in Table 1.

### Associated symptoms and signs

The additional features that were ascertained in our CMT patient cohort are listed in Table 2. A total of 65% (32/49) of the patients presented two or more additional symptoms, 23% (11/49) had one, and 12% (6/49) expressed no additional features (Table 2).

#### Hypertrophic cauda equina or nerve roots

**Figure 1:**
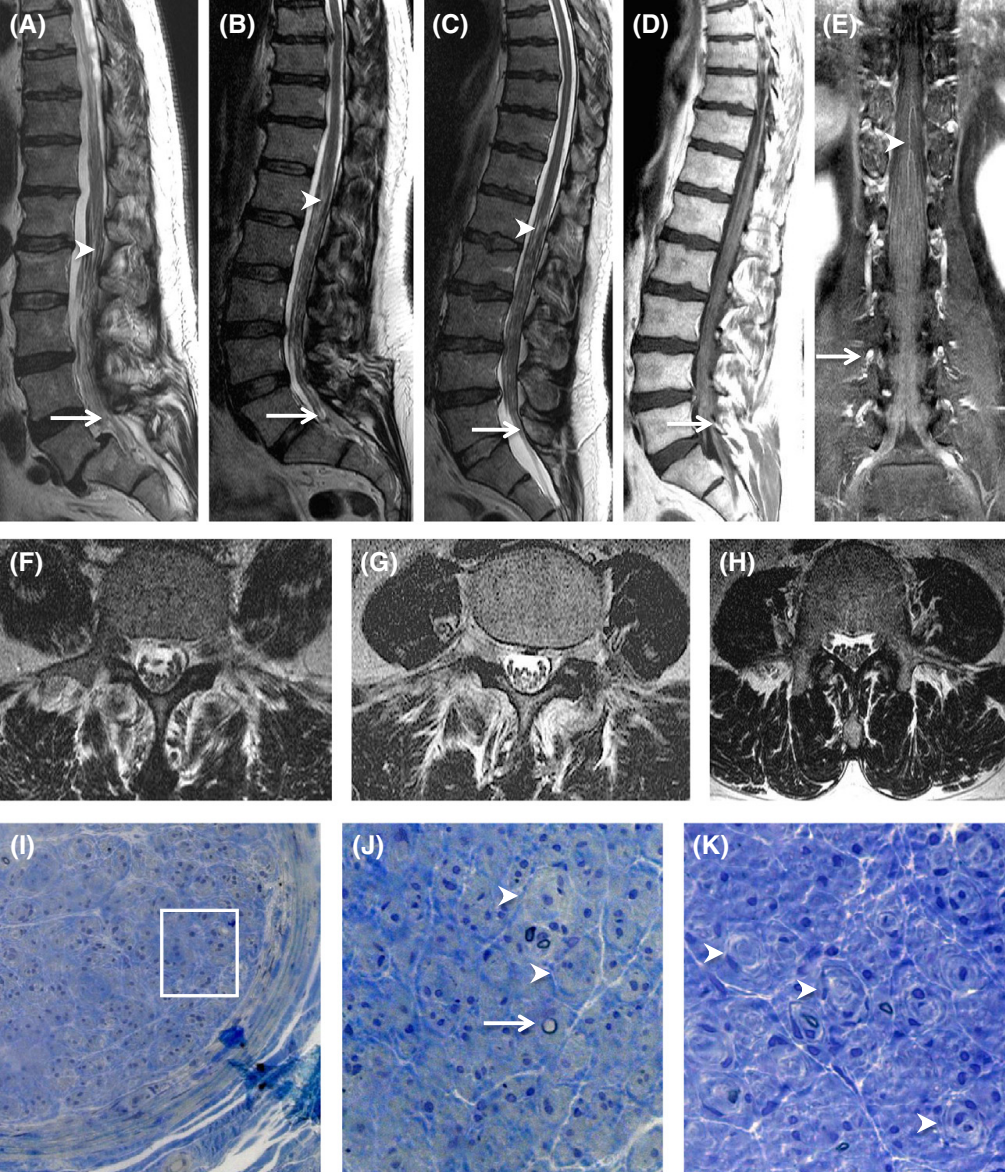
Thoraco‐lumbo‐sacral MRI and sural nerve biopsies in three CMT1B patients with *MPZ*‐mutation and hypertrophic cauda equina/nerve roots. (A–C) T2‐weighted sagittal sections of the thoracal, lumbar, and sacral spinal column in patients 6 (A), 4 (B), and 2 (C), indicating the thickened nerve roots of the cauda equina (arrows). This is also shown for patient 2 at a T1‐weighted sagittal section (D) and a T1‐weighted coronal section (E). Arrow heads show congested intraspinal vessels. (F–H) T2‐weighted axial sections at the level of the fourth lumbar vertebral body (L4) of patients 6 (F), 4 (G) and 2 (H), showing extensive hypertrophic nerve roots of the cauda equina inside the spinal column. (I–K) Nerve Biopsy pictures of patients 6 (I, J) and 2 (K) with (J) being a magnification of (I). Arrow heads show onion bulb formations, the arrow points toward a remaining hypomyelinated nerve fiber. Notice the extensive loss of the large myelinated fibers.

MRI of the spine revealed a hypertrophic cauda equina in three patients with a mutation in *MPZ* (CMT1B): in a brother and sister (patients 2 and 4) belonging to family 1 and in a third unrelated patient with a distinct *MPZ* mutation (patient 6) (Tables 1 and 2; Fig. 1). The affected father of patients 2 and 4 (patient 3) was not available for MRI examination and patient 10, who harbored the same *MPZ* mutation as patient 6, could not undergo the examination due to marked respiratory insufficiency. We did not find hypertrophic nerve roots or cauda fascicles in the two patients with a mutation in *LITAF* (CMT1C), who underwent a cervical (patient 12) or lumbar MRI (patient 13). In the other study patients, a spinal MRI was not performed. Hypertrophy of cranial nerves was not additionally revealed on brain MRI in patients 2 and 6, neither in the other patients (patients 1, 5 12, 22) in whom a brain MRI was available (Table 2).

##### Clinical presentation of the three patients with MPZ mutation and hypertrophic nerve roots

Patients 2 and 4 (brother and sister) had disease onset in childhood (patient 2) and at 38 years of age (patient 4) and patient 6 at 62 years (Table 1). They presented typical symptoms and signs of CMT. Additional symptoms were scoliosis, anisocoric, and tonic pupils in patient 2, bilateral carpal tunnel syndrome (CTS), tonic pupils, and scoliosis in patient 4, and a sleep apnea syndrome in patient 6. Pupils could not be evaluated in patient 6 due to previous bilateral cataract surgery. Patient 4 complained of lower back pains and patient 6 of occasional lumbar pain radiating in the legs, which can be explained by the hypertrophic lumbar nerve roots.

NCVs were within the range of demyelinating CMT with motor NCV in the upper limbs of 19 m/sec (patient 2), 26 m/sec (patient 4), and 18 m/sec (patient 6). Liquor analysis in patients 2 and 6 revealed elevated protein levels (1.45 g/L and 0.46 g/L, respectively, normal value 0.15–0.45 g/L), and was not performed in patient 4. Spinal MRI in the three patients demonstrated bilaterally multiple enlarged nerve roots within the spinal column at the lumbar and sacral level (Fig. 1A–H), leading to congested intraspinal vessels (Fig. 1A–C and E). Sural nerve biopsies showed a prominent loss of large myelinated fibers (Fig. 1I–K), as well as Schwann cells forming multiple layers of myelin sheaths around lost fibers leading to onion bulb formations (Fig. 1J and K).

Our revision of the literature revealed the occurrence of hypertrophic nerve roots at the cervical and/or lumbar spine in association with *MPZ* mutations: c.306delA in two patients and c.167G>A in another two cases, as well as *PMP22* gene alterations in five cases, of which three patients with *PMP22* duplication, one with a homozygous *PMP22* duplication, and one carrying a *PMP22* point mutation (Appendix S1). In our study, we added two distinct *MPZ* mutations associated with this feature (Table 1). Furthermore, hypertrophy of the cranial nerves has also been described in two patients with a *MPZ* mutation and in another two with a *PMP22* gene deletion (Appendix S1).

#### Restless legs syndrome

Restless legs syndrome (RLS) was present in nine patients of our cohort (18%), five females and four males, aged 27, 32, 33, 39, 51, 54, 56, 63, and 65 years. They carried a mutation in the *MPZ*,* GJB1*, or *MFN2* gene (one patient each), in *LITAF* (two patients), or a *PMP22* duplication (two patients) or deletion (two patients). CMT patients with RLS had CMTNS2 scores ranging from mainly severe (patient 1, 40, 45, 13), to moderate (patient 17) or mild neuropathy (patient 12) (Table 1).

In the literature, RLS has been associated with three different CMT genes (Appendix S1), among them also the genes that we have identified in our patient group, except for the *LITAF* and *MFN2* genes that were novel findings in our study.

#### Respiratory insufficiency and scoliosis

In patient 10 with CMT2‐I/J caused by a *MPZ* mutation, a severe restrictive respiratory insufficiency with vital capacity of 1.2 L (36.8% of the theoretical value) leading to severe dyspnea and orthopnea was diagnosed at the age of 48 years. Respiratory symptoms started at least 6 years before. Neurological examination at 48 years revealed distally pronounced weakness and sensory loss, pes cavus, clawed hands, and fasciculations at the extremities. She reported onset of CMT symptoms between 20 and 25 years of age. Due to pronounced orthopnea, the patient was not able to lie down, slept in sitting position, and needed a CPAP mask for sufficient oxygenation. Interestingly, patient 6 with the same R98H mutation in MPZ showed no signs of respiratory compromise (Table 2). Furthermore, we diagnosed respiratory insufficiency with a vital capacity of 1.91 L (54% of the theoretical value) at 25 years of age in patient 30 harboring a *PMP22* duplication. CMTNS2 scores were severe in both patients (Table 1). They did not have a scoliosis as a possible cause of respiratory problems.

Mild scoliosis was noted in 14 patients and in one patient (patient 41, CMT1A), scoliosis was severe, necessitating a surgical correction. Three had a mutation in *MPZ* (CMT1B), eight had a *PMP22* duplication, one a *PMP22* point mutation, one a *PMP22* deletion, one carried a *GJB1* mutation, and one patient had a mutation in *LITAF* (Table 2).

Literature findings revealed that respiratory insufficiency and scoliosis are frequent additional features in many different CMT subtypes including those that we have detected (Appendix S1).

#### Pupillary abnormalities

Pupillary abnormalities were noticed in three of our patients with a *MPZ* mutation. Two of them had the CMT1B phenotype (patients 2 and 4) and showed tonic pupils and/or anisocoria. The third patient, with CMT2 I/J (patient 5), presented tonic pupils, anisocoria, and mydriasis (Table 2). In patients 3 and 6, reactions to light were not measurable because of a previous bilateral cataract surgery. CMTNS2 scores in our patients with pupillary abnormalities were in the range of mild (patients 4 and 5) or moderate (patient 2) neuropathy severity (Table 1).

Our literature review revealed that pupillary abnormalities have been reported with mutations in 10 different CMT‐causing genes (Appendix S1). Most frequently occurring abnormalities are tonic pupils [*PMP22, MPZ, GJB1,* SH3 domain, and tetratricopeptide repeat domain 2 (*SH3TC2*) gene*,* set‐binding factor 1 (*SBF1*) gene], or an impaired pupillary reaction [transient receptor potential cation channel subfamily V, member 4 (*TRPV4*) gene, NMYC downstream‐regulated gene 1 (*NDRG1*) gene]. Furthermore, anisocoria [*PMP22, MPZ, MFN2, SH3TC2*; FYVE, RhoGEF, and PH domain‐containing protein 4 (*FGD4*) gene], miosis (*PMP22, MPZ*) and mydriasis (*MPZ*) have been described previously.

#### Cold‐induced hand cramps

In our cohort, cold‐induced hand cramps were indicated during history taking by one patient with *MPZ* mutation (CMT1B, patient 7), two cases with *PMP22* duplication (patients 39, 40), one patient with *PMP22* deletion (patient 24), and one with a mutation in *MFN2* (patient 48) (Table 2).

In contrast to our findings, literature data revealed this symptom only in association with a mutation in the glycyl‐tRNA synthetase (*GARS*) gene (Appendix S1).

#### Focal segmental glomerular sclerosis

In one of the two patients with histologically proven FSGS and a polyneuropathy, we identified a pathogenic mutation in the *INF2* gene (patient 47). Literature findings confirmed that renal problems and FSGS in particular in CMT patients occur so far exclusively in association with mutations in *INF2*. In addition, deafness, cerebral white matter hyperintensities, and enlarged ventricles have been described in association with *INF2* mutations (Appendix S1). These features were, however, not present in our patient.

### Summary of associated features in CMT

Considering our study cohort and literature data, we detected more than 80 different additional symptoms or signs associated with CMT (Appendix S1, Table 3). Table 3 summarizes the new findings in our cohort compared to the literature (Table 3).

**Table 3:** Additional symptoms identified in our CMT cohort and occurrence in the literature

Autonomous: U urgency/incontinence	*PMP22*,*MPZ*,**MFN2**,LRSAM1,TRPV4,SBF1
Autonomous: Postural hypotension	**PMP22**,*MPZ*,**LITAF**
Autonomous: Hyperhidrosis	**PMP22**,**MPZ,**MFN2,GDAP1
Bulbar (dysphagia, dysarthria, cranial nerve involvement of IX/X)	*PMP22*,MPZ,GJB1,EGR2,MFN2,GDAP1,NEFL,SH3TC2,MTMR2,TFG,NDRG1,IFRD1,TRPV4,SBF1
Cognitive impairment	*PMP22*,**MPZ**,GJB1,MFN2,INF2,DYNC1H1,NEFL,DNM2,**LITAF**,SLC12A6,HK1,AIFM1,SBF1
Cold‐induced hand cramps	**PMP22**,**MPZ**,**MFN2**,GARS
Carpal tunnel syndrome	**PMP22**,**MPZ**,PRX,**LITAF**,FIG 4,TRPV4,FBLN5
Deafness	*PMP22*,MPZ,GJB1,PRX,EGR2,MFN2,PDK3,SURF1,INF2,NEFL,AARS,LRSAM1,SH3TC2,TFG,LITAF,SBF2,NDRG1,IFRD1 TRPV4,AIFM1,PRPS1
Early onset:	PMP22,*MPZ*,GJB1,PRX,EGR2,MFN2,PDK3,GDAP1,INF2,DYNC1H1,NEFL,TRIM2,KARS,SH3TC2,FIG 4,DNM2,MTMR2 FGD4,NDRG1,TRPV4,SCL12A6,AIFM1
Eye: Pupillary	PMP22,*MPZ*,GJB1,EGR2,MFN2,SH3TC2,FGD4,NDRG1,TRPV4,SBF1
Facial weakness	*PMP22*,MPZ,EGR2,MFN2,GDAP1,NEFL,SH3TC2,FIG 4,DNM2,MTMR2,NDRG1,TRPV4,TRG,SLC12A6,HK1,SBF1
Fasciculations	PMP22,**MPZ**,PRX,EGR2,NEFL,HINT1,LRSAM1,SH3TC2,RAB7,TFG,HSPB1,AIFM1
Hand deformities	*PMP22*,*MPZ*,PRX,EGR2,GDAP1,INF2,LMNA,NEFL,SH3TC2,FIG 4,MTMR2,SBF2,NDRG1,HSPB1,HK1,FBLN5
MRI, brain: White matter involvement	PMP22,*MPZ*,GJB1,MFN2,INF2,SH3TC2,NDRG1,SLC12A6,AIFM1
MRI, spinal: Hypertrophic nerve roots	PMP22,*MPZ*
Pain	*PMP22*,*MPZ*,**GJB1**,*MFN2*,GDAP1,INF2,DYNC1H1,MARS,HARS,SH3TC2,RAB7,FIG 4,TFG,*LITAF*,SBF2,HSPB1
Paresthesia	*PMP22*,*MPZ*,*GJB1*,PRX,**MFN2**,**NEFL**,FIG 4,DNM2,TFG,*LITAF*,HSJ1,MED25,FBLN5
Renal problems	*INF2*
Respiratory insufficiency	**PMP22**,*MPZ*,PRX,EGR2,MFN2,GDAP1,SH3TC2,FIG 4,MTMR2,TFG,TRPV4,FBLN5
Restless legs syndrome	*PMP22*,*MPZ*,*GJB1*,**MFN2**,**LITAF**
Skeletal: Congenital hip dysplasia	*PMP22*,EGR2
Skeletal: Hyperkyphosis	**INF2**,PRX,MFN2,GDAP1,SLC12A6
Skeletal: Scoliosis	*PMP22*,*MPZ*,*GJB1*,PRX,EGR2,MFN2,GDAP1,SURF1,INF2,LMNA,NEFL,GARS,SH3TC2,RAB7,FIG 4,MTMR2,*LITAF*,FGD4 SBF2,NDRG1,HSPB8,TRPV4,HK1,FBLN5,SBF1
Skeletal: Ulnar deviation of hands	PMP22,**INF2**
Tremor	*PMP22*,*MPZ*,*GJB1*,PRX,EGR2,*MFN2*,PDK3,INF2,DYNC1H1,NEFL,SH3TC2,FIG 4,TFG,*LITAF*,FGD4,NDRG1,IFRD1,TRPV4 SCL12A6,HK1
Upper limb predominant	**LITAF**,GARS

Bold: new findings in current patient cohort; italics and underlined: findings in current study that have been reported previously; not underlined, not italics and not bold: literature findings. CMT, Charcot–Marie–Tooth neuropathy; U, urinary. Additional features are listed in alphabetical order.

Regarding the additional features of all CMT‐causing genes described in the literature, many additional features were reported in association with many distinct CMT‐causing genes, for example, vocal cord involvement, tremor, scoliosis, hand deformities (claw hands and/or finger contractures), deafness, cognitive impairment, bulbar symptoms, fasciculations, facial weakness, early proximal weakness, pain, paresthesias, early onset CMT, pupillary abnormality, ophthalmoparesis, respiratory failure/distress, SAS, dysmorphic features, central nervous system/cranial nerve involvement, brain imaging abnormality, white matter involvement, severe slow NCV, and kyphosis/lordosis (Table 3, Appendix S1).

On the contrary, other additional symptoms were described in a single patient or family only and in association with only one gene, such as optic neuritis, chronic vomiting, bowel dysfunction, mutilating arthropathy, nocturnal vomiting, hyperkeratosis, myokymia, cardiac insufficiency and cardiomyopathy, involuntary movements, edema, self‐abusive behavior, acrocyanosis, lactic acidosis under fasting conditions, and slowed emptying of stomach (Appendix S1).

### Link between additional features and gene function?

We studied whether the occurrence of certain additional symptoms might be related to mutations in genes that express a similar function. We considered as distinct functional groups (Azzedine et al. 2012; Timmerman et al. 2013): genes expressing a role in myelination, mitochondrial functioning, cytoskeletal stability and motor proteins, RNA and protein metabolism, protein folding, membrane traffic, transcription regulation, channel or transporter and other/unknown (Appendix S1). However, we did not find a correlation between the additional features and the function of the causative genes.

### Mutations identified in our cohort compared with the same mutations reported in the literature

We compared the clinical phenotype and in particular the associated features related to the mutations identified in our cohort with literature findings on the same mutations (Appendix S2). If clinical data were not available in addition to the mutation in the literature, the respective publication was not included in the Appendix S2.

## Discussion

We showed that the majority of CMT patients presented one or more feature in addition to the classical CMT phenotype. Several additional features revealed by our study, such as hypertrophic nerve roots and RLS, seem to be underestimated. Their occurrence broaden the clinical spectrum and genotype–phenotype associations in CMT.

### Methodological issues of our study and future perspectives

Due to the partly retrospective nature of our study, some data such as MRI images or nerve biopsies were lacking in some patients. Furthermore, although our overall patient cohort was large considering the rarity of the disease, some of the subgroups, such as CMT1F and CMT2A2, were small. However, we additionally included in our study all data of the currently available literature (in total 280 papers, Table 3, Appendices S1 and S2), in order to obtain more data and larger subgroups. Our results are very interesting, but prospective larger multicenter studies will be necessary in the future to confirm our findings.

### Hypertrophic nerve roots as additional finding in patients with CMT

Hypertrophic nerve roots can occur in hereditary disorders (e.g., CMT1, CMT3, neurofibromatosis), infectious or inflammatory neuropathies, neoplastic nerve disorders, and diverse acquired diseases such as amyloidosis (Ginsberg et al. 1995). Hypertrophied nerve roots in CMT have been reported previously by using pathological, ultrasonographic, and MRI examinations (Andermann et al. 1962; Symonds and Blackwood 1962; Marchini et al. 2009; Sugimoto et al. 2013). Thus, the presence of hypertrophic nerve roots in CMT is not new, and is probably often not reported since there is rarely a compelling reason to perform MRI in patients with CMT. So far, two CMT‐causing genes, *PMP22* and *MPZ,* have been related to this feature (Butefisch et al. 1999; Kleopa et al. 2002; Simonati et al. 2002; Marchini et al. 2009) (Table 3, Appendix S1). Also in our cohort, three CMT1B patients with a *MPZ* mutation presented a hypertrophic cauda equina. In contrast to another case in which the hypertrophic nerve roots and cauda resulted in a compression with severe symptoms and improvement by surgery (Kleopa et al. 2002), our patients did not show signs of medullar compression. Interestingly, we observed hypertrophic nerve roots in two relatives with CMT1B (brother and sister, family 1) as well as in a third unrelated individual. The two mutations in the *MPZ* gene that were associated with this feature in our study (Table 1), have been described in the literature before; however, in those cases, hypertrophic nerve roots were not reported (Appendix S2). Hypertrophic nerve roots in CMT1B‐patients were mainly described in isolated cases (Kleopa et al. 2002; Simonati et al. 2002) and in only one family comprising a father and his two affected daughters carrying the Val102 fs mutation in *MPZ* (Marchini et al. 2009). Hypertrophic nerve roots caused by mutations in *MPZ* were not only found in association with the CMT1B phenotype, but also with congenital hypomyelinating neuropathy (CHN) (Simonati et al. 2002) and Dejerine–Sottas neuropathy (DSS) (Kleopa et al. 2002). Most authors presume that the thickening of nerve roots is due to a Schwann cell proliferation in the context of consistent de‐ and remyelination over the years (Kleopa et al. 2002; Simonati et al. 2002). This explanation is further strengthened by the finding that the number of onion bulb formations in sural nerve biopsies, indicating active de‐ and remyelination, correlated with the occurrence of spinal nerve root enhancement and thickening on lumbosacral MRI in CMT patients (Cellerini et al. 2000).

### RLS as additional symptom in CMT

RLS was present in 18% of our cohort, which was higher compared to the prevalence of 9.8% in the general population above 65 years of age (Rothdach et al. 2000). In our cohort, five females (19% of all females, aged 32, 51, 54, 56, and 65 years) and four males (18% of all males, aged 27, 33, 39, and 63 years) were affected. In contrast to these quite similar numbers, women in the general population were found to be affected twice as often as men (13.9% vs. 6.1%) (Rothdach et al. 2000) and also female CMT patients showed RLS more frequently than male CMT patients in previous studies (Boentert et al. 2010, 2014). The prevalence of RLS has previously been shown to be significantly increased in hereditary neuropathies, including CMT1, CMT2, CMTX, and HNPP (Hattan et al. 2009). Since RLS is frequently associated with peripheral neuropathy in general, our findings were, however, to be expected (Hattan et al. 2009). In our cohort, eight out of nine patients with RLS harbored demyelinating subtypes with mutations in the *MPZ* (*n* = 1), *GJB1* (*n* = 1), or *LITAF* gene (*n* = 2) or a *PMP22* duplication (*n* = 2) or deletion (*n* = 2) and one patient with RLS displayed an axonal neuropathy with *MFN2* gene mutation (Table 2). In the literature, RLS was described in cohorts consisting of CMT patients with *PMP22* duplication and *MPZ* or *GJB1* gene mutation (Appendix S1). Female CMT patients were also found to be more severely affected by RLS than men (Boentert et al. 2010, 2014). In our cohort, four patients suffering from RLS had severe (two male and two female, patient 1, 40, 45, 13), one moderate (male, patient 17), and one mild neuropathy (male, patient 12) (Table 1). One hypothesis is that axonal atrophy increases axonal excitability in primary sensory units of leg muscles which results in creeping sensations (Iannaccone et al. 1995). As a consequence, this sensory input in the central nervous system may lead to the oscillatory leg movements typical for RLS (Iannaccone et al. 1995). This hypothesis links axonal atrophy with RLS. In CMT patients, axonal damage may occur in axonal subtypes as well as in demyelinating subtypes, where it develops consequently to the loss of myelin (Boentert et al. 2010). This might explain how CMT may predispose to RLS.

### Pupillary abnormalities as additional feature in CMT patients

In our cohort, pupillary abnormalities were seen in three out of 11 patients with a *MPZ* gene mutation (27%): two CMT1B patients belonging to one family (patients 2 and 4) and one isolated CMT2‐I/J patient (patient 5) (Tables 1 and 2). Their pupil changes became apparent at 51, 41, and 23 years of age, respectively. In another eight patients with *MPZ* gene mutation, one CMT2 and seven CMT1, we did not detect pupil changes (Tables 1 and 2). Families and isolated CMT patients have been described with pupillary abnormalities harboring mutations in 10 different CMT‐causing genes, including the *MPZ* gene (Table 3, Appendix S1). Certain *MPZ* mutations were found to be particularly associated with pupil abnormalities, such as the Thr124Met mutation leading to CMT2‐I/J in most patients. This mutation is associated with a distinct clinical phenotype including late onset in the fourth or fifth decade, pupillary abnormalities, shooting pains, deafness, explicit sensory disturbances, rapid progression, various autonomic system involvement, and respiratory insufficiency (De Jonghe et al. 1999; Stojkovic et al. 2003). Patient 5 of our cohort also showed the CMT2‐I/J phenotype with pupillary abnormalities associated with the novel *MPZ* mutation Gly123Ala (Table 1). She complained of painful paresthesia in the lower limbs, hands, and face, compatible with the lancinating pains described in patients with the Thr124Met mutation. In contrast, her disease symptoms already began in the third decade, disease progression was rather slow, and no further associated features such as deafness or autonomic system involvement were present (Table 2).

## Conclusions

We conclude that additional features are present in the majority of CMT patients. In case of rather specific features, occurring in association with only one (e.g., renal problems) or a few different CMT genes (e.g., hypertrophic nerve roots, Table 3), they might be used to guide genetic diagnosis by performing one or only a few single‐gene analyses. However, many other additional symptoms, such as hearing impairment or cognitive involvement, can present in association with a large number of distinct genes (Table 3, Appendix S1), which hampers a gene search based on the additional features. Therefore, in these cases, it might diagnostically be more efficient to directly perform novel genetic techniques (WES), in which a large number of genes can be tested at the same time. However, aside from additional clinical features, the mode of inheritance, NCVs, and pathological examination of nerve biopsies also remain important clues to molecular diagnosis in CMT.

## Conflict of Interest

The authors declare that they have no conflicts of interest.

## Supporting information


**Appendix S1.** Additional symptoms and features in patients with CMT reported in the literature.

**Appendix S2.** Phenotypic comparison of mutations found in our cohort with the literature.

## References

[brb3451-bib-0120] Ananth, U. , Athena Diagnostics Inc. 1999. Personal data. http://www.molgen.ua.ac.be/cmtmutations/Mutations/Mutations.cfm

[brb3451-bib-0001] Andermann, F. , D. L. Lloyd‐Smith , H. Mavor , and G. Mathieson . 1962 Observations on hypertrophic neuropathy of Dejerine and Sottas. Neurology 12:712–724. 13861139 10.1212/wnl.12.10.712

[brb3451-bib-0002] Azzedine, H. , J. Senderek , C. Rivolta , and R. Chrast . 2012 Molecular genetics of charcot‐marie‐tooth disease: from genes to genomes. Mol. Syndromol. 3:204–214. 23293578 10.1159/000343487 PMC3531925

[brb3451-bib-0003] Boentert, M. , R. Dziewas , A. Heidbreder , S. Happe , I. Kleffner , S. Evers , et al. 2010 Fatigue, reduced sleep quality and restless legs syndrome in Charcot‐Marie‐Tooth disease: a web‐based survey. J. Neurol. 257:646–652. 19937049 10.1007/s00415-009-5390-1 PMC3128702

[brb3451-bib-0004] Boentert, M. , K. Knop , C. Schuhmacher , B. Gess , A. Okegwo , and P. Young . 2014 Sleep disorders in Charcot‐Marie‐Tooth disease type 1. J. Neurol. Neurosurg. Psychiatry 85:319–325. 23704315 10.1136/jnnp-2013-305296

[brb3451-bib-0005] Bort, S. , E. Nelis , V. Timmerman , T. Sevilla , A. Cruz‐Martinez , F. Martinez , et al. 1997 Mutational analysis of the MPZ, PMP22 and Cx32 genes in patients of Spanish ancestry with Charcot‐Marie‐Tooth disease and hereditary neuropathy with liability to pressure palsies. Hum. Genet. 99:746–754. 9187667 10.1007/s004390050442

[brb3451-bib-0006] Boyer, O. , F. Nevo , E. Plaisier , B. Funalot , O. Gribouval , G. Benoit , et al. 2011 INF2 mutations in Charcot‐Marie‐Tooth disease with glomerulopathy. N. Engl. J. Med. 365:2377–2388. 22187985 10.1056/NEJMoa1109122

[brb3451-bib-0007] Butefisch, C. , L. Gutmann , and L. Gutmann . 1999 Compression of spinal cord and cauda equina in Charcot‐Marie‐Tooth disease type 1A. Neurology 52:890–891. 10078755 10.1212/wnl.52.4.890

[brb3451-bib-0008] Cellerini, M. , S. Salti , V. Desideri , and G. Marconi . 2000 MR imaging of the cauda equina in hereditary motor sensory neuropathies: correlations with sural nerve biopsy. AJNR Am. J. Neuroradiol. 21:1793–1798. 11110529 PMC7974297

[brb3451-bib-0170] Chance, P. F. , M. K. Alderson , K. A. Leppig , M. W. Lensch , N. Matsunami , B. Smith , et al. 1993 DNA deletion associated with hereditary neuropathy with liability to pressure palsies. Cell 72:143–151. 8422677 10.1016/0092-8674(93)90058-x

[brb3451-bib-0009] Chung, K. W. , S. B. Kim , K. D. Park , K. G. Choi , J. H. Lee , H. W. Eun , et al. 2006 Early onset severe and late‐onset mild Charcot‐Marie‐Tooth disease with mitofusin 2 (MFN2) mutations. Brain 129:2103–2118. 16835246 10.1093/brain/awl174

[brb3451-bib-0010] Davis, C. J. , W. G. Bradley , and R. Madrid . 1978 The peroneal muscular atrophy syndrome: clinical, genetic, electrophysiological and nerve biopsy studies. I. Clinical, genetic and electrophysiological findings and classification. J. Genet. Hum. 26:311–349. 752065

[brb3451-bib-0011] De Jonghe, P. , V. Timmerman , C. Ceuterick , E. Nelis , E. De Vriendt , A. Lofgren , et al. 1999 The Thr124Met mutation in the peripheral myelin protein zero (MPZ) gene is associated with a clinically distinct Charcot‐Marie‐Tooth phenotype. Brain 122(Pt 2):281–290. 10071056 10.1093/brain/122.2.281

[brb3451-bib-0012] Eggers, S. D. , S. C. Keswani , G. Melli , and D. R. Cornblath . 2004 Clinical and genetic description of a family with Charcot‐Marie‐Tooth disease type 1B from a transmembrane MPZ mutation. Muscle Nerve 29:867–869. 15170620 10.1002/mus.20034

[brb3451-bib-0013] Fabrizi, G. M. , M. Pellegrini , C. Angiari , T. Cavallaro , A. Morini , F. Taioli , et al. 2006 Gene dosage sensitivity of a novel mutation in the intracellular domain of P0 associated with Charcot‐Marie‐Tooth disease type 1B. Neuromuscul. Disord. 16:183–187. 16488608 10.1016/j.nmd.2006.01.006

[brb3451-bib-0014] Gabreels‐Festen, A. A. , J. E. Hoogendijk , P. H. Meijerink , F. J. Gabreels , P. A. Bolhuis , S. van Beersum , et al. 1996 Two divergent types of nerve pathology in patients with different P0 mutations in Charcot‐Marie‐Tooth disease. Neurology 47:761–765. 8797476 10.1212/wnl.47.3.761

[brb3451-bib-0015] Gerding, W. M. , J. Koetting , J. T. Epplen , and C. Neusch . 2009 Hereditary motor and sensory neuropathy caused by a novel mutation in LITAF. Neuromuscul. Disord. 19:701–703. 19541485 10.1016/j.nmd.2009.05.006

[brb3451-bib-0016] Ginsberg, L. , A. D. Platts , and P. K. Thomas . 1995 Chronic inflammatory demyelinating polyneuropathy mimicking a lumbar spinal stenosis syndrome. J. Neurol. Neurosurg. Psychiatry 59:189–191. 7629539 10.1136/jnnp.59.2.189 PMC486000

[brb3451-bib-0017] Harding, A. E. , and P. K. Thomas . 1980 The clinical features of hereditary motor and sensory neuropathy types I and II. Brain 103:259–280. 7397478 10.1093/brain/103.2.259

[brb3451-bib-0018] Hattan, E. , C. Chalk , and R. B. Postuma . 2009 Is there a higher risk of restless legs syndrome in peripheral neuropathy? Neurology 72:955–960. 19038854 10.1212/01.wnl.0000336341.72621.db

[brb3451-bib-0019] Hattori, N. , M. Yamamoto , T. Yoshihara , H. Koike , M. Nakagawa , H. Yoshikawa , et al. 2003 Demyelinating and axonal features of Charcot‐Marie‐Tooth disease with mutations of myelin‐related proteins (PMP22, MPZ and Cx32): a clinicopathological study of 205 Japanese patients. Brain 126:134–151. 12477701 10.1093/brain/awg012

[brb3451-bib-0021] Iannaccone, S. , M. Zucconi , P. Marchettini , L. Ferini‐Strambi , R. Nemni , A. Quattrini , et al. 1995 Evidence of peripheral axonal neuropathy in primary restless legs syndrome. Mov. Disord. 10:2–9. 7885351 10.1002/mds.870100103

[brb3451-bib-0022] Kleopa, K. A. , L. N. Sutton , J. Ong , G. Tennekoon , and A. E. Telfeian . 2002 Conus medulla‐cauda compression from nerve root hypertrophy in a child with Dejerine‐Sottas syndrome: improvement with laminectomy and duraplasty. Case report. J. Neurosurg. 97:244–247. 12296688 10.3171/spi.2002.97.2.0244

[brb3451-bib-0300] Lupski, J. R. , R. M. de Oca‐Luna , S. Slaugenhaupt , L. Pentao , V. Guzzetta , B. J. Trask , et al. 1991 DNA duplication associated with Charcot‐Marie‐Tooth disease type 1A. Cell 66:219–232. 1677316 10.1016/0092-8674(91)90613-4

[brb3451-bib-0023] Mademan, I. , T. Deconinck , A. Dinopoulos , T. Voit , U. Schara , K. Devriendt , et al. 2013 De novo INF2 mutations expand the genetic spectrum of hereditary neuropathy with glomerulopathy. Neurology 81:1953–1958. 24174593 10.1212/01.wnl.0000436615.58705.c9

[brb3451-bib-0024] Marchini, C. , S. Z. Marsala , M. Bendini , F. Taioli , G. Damante , I. R. Lonigro , et al. 2009 Myelin protein zero Val102 fs mutation manifesting with isolated spinal root hypertrophy. Neuromuscul. Disord. 19:849–852. 19906531 10.1016/j.nmd.2009.09.004

[brb3451-bib-0025] Mersiyanova, I. V. , A. V. Perepelov , A. V. Polyakov , V. F. Sitnikov , E. L. Dadali , R. B. Oparin , et al. 2000 A new variant of Charcot‐Marie‐Tooth disease type 2 is probably the result of a mutation in the neurofilament‐light gene. Am. J. Hum. Genet. 67:37–46. 10841809 10.1086/302962 PMC1287099

[brb3451-bib-0026] Murphy, S. M. , D. N. Herrmann , M. P. McDermott , S. S. Scherer , M. E. Shy , M. M. Reilly , et al. 2011 Reliability of the CMT neuropathy score (second version) in Charcot‐Marie‐Tooth disease. J. Peripher. Nerv. Syst. 16:191–198. 22003934 10.1111/j.1529-8027.2011.00350.x PMC3754828

[brb3451-bib-0027] Numakura, C. , C. Lin , T. Ikegami , P. Guldberg , and K. Hayasaka . 2002 Molecular analysis in Japanese patients with Charcot‐Marie‐Tooth disease: DGGE analysis for PMP22, MPZ, and Cx32/GJB1 mutations. Hum. Mutat. 20:392–398. 12402337 10.1002/humu.10134

[brb3451-bib-0028] van Paassen, B. W. , A. J. van der Kooi , K. Y. van Spaendonck‐Zwarts , C. Verhamme , F. Baas , and M. de Visser . 2014 PMP22 related neuropathies: Charcot‐Marie‐Tooth disease type 1A and hereditary neuropathy with liability to pressure palsies. Orphanet J. Rare Dis. 9:38. 24646194 10.1186/1750-1172-9-38 PMC3994927

[brb3451-bib-0400] Raeymaekers, P. , V. Timmerman , E. Nelis , P. De Jonghe , J. E. Hoogendijk , F. Baas , et al. 1991 Duplication in chromosome 17p11.2 in Charcot‐Marie‐Tooth neuropathy type 1a (CMT 1a). The HMSN Collaborative Research Group. Neuromuscul. Disord. 1:93–97. 1822787 10.1016/0960-8966(91)90055-w

[brb3451-bib-0029] Roos, A. , J. Weis , R. Korinthenberg , H. Fehrenbach , M. Hausler , S. Zuchner , et al. 2015 Inverted formin 2‐related Charcot‐Marie‐Tooth disease: extension of the mutational spectrum and pathological findings in Schwann cells and axons. J. Peripher. Nerv. Syst. 20:52–59. 25676889 10.1111/jns.12106

[brb3451-bib-0030] Rossor, A. M. , J. M. Polke , H. Houlden , and M. M. Reilly . 2013 Clinical implications of genetic advances in Charcot‐Marie‐Tooth disease. Nat. Rev. Neurol. 9:562–571. 24018473 10.1038/nrneurol.2013.179

[brb3451-bib-0031] Rothdach, A. J. , C. Trenkwalder , J. Haberstock , U. Keil , and K. Berger . 2000 Prevalence and risk factors of RLS in an elderly population: the MEMO study. Memory and morbidity in Augsburg elderly. Neurology 54:1064–1068. 10720275 10.1212/wnl.54.5.1064

[brb3451-bib-0032] Schröder, J. M. 2001 Pathology of peripheral nerves. An atlas of structural and molecular pathological changes.

[brb3451-bib-0033] Simonati, A. , G. M. Fabrizi , F. Taioli , A. Polo , R. Cerini , and N. Rizzuto . 2002 Dejerine‐Sottas neuropathy with multiple nerve roots enlargement and hypomyelination associated with a missense mutation of the transmembrane domain of MPZ/P0. J. Neurol. 249:1298–1302. 12242557 10.1007/s00415-002-0810-5

[brb3451-bib-0034] Skre, H. 1974 Genetic and clinical aspects of Charcot‐Marie‐Tooth's disease. Clin. Genet. 6:98–118. 4430158 10.1111/j.1399-0004.1974.tb00638.x

[brb3451-bib-0035] Stojkovic, T. , J. de Seze , O. Dubourg , M. C. Arne‐Bes , S. Tardieu , J. C. Hache , et al. 2003 Autonomic and respiratory dysfunction in Charcot‐Marie‐Tooth disease due to Thr124Met mutation in the myelin protein zero gene. Clin. Neurophysiol. 114:1609–1614. 12948789 10.1016/s1388-2457(03)00159-7

[brb3451-bib-0036] Street, V. A. , G. Meekins , H. P. Lipe , W. K. Seltzer , G. T. Carter , G. H. Kraft , et al. 2002 Charcot‐Marie‐Tooth neuropathy: clinical phenotypes of four novel mutations in the MPZ and Cx 32 genes. Neuromuscul. Disord. 12:643–650. 12207932 10.1016/s0960-8966(02)00021-4

[brb3451-bib-0037] Sugimoto, T. , K. Ochi , N. Hosomi , T. Takahashi , H. Ueno , T. Nakamura , et al. 2013 Ultrasonographic nerve enlargement of the median and ulnar nerves and the cervical nerve roots in patients with demyelinating Charcot‐Marie‐Tooth disease: distinction from patients with chronic inflammatory demyelinating polyneuropathy. J. Neurol. 260:2580–2587. 23821028 10.1007/s00415-013-7021-0

[brb3451-bib-0038] Symonds, C. P. , and W. Blackwood . 1962 Spinal cord compression in hypertrophic neuritis. Brain 85:251–260.

[brb3451-bib-0039] Timmerman, V. , V. E. Clowes , and E. Reid . 2013 Overlapping molecular pathological themes link Charcot‐Marie‐Tooth neuropathies and hereditary spastic paraplegias. Exp. Neurol. 246:14–25. 22285450 10.1016/j.expneurol.2012.01.010

[brb3451-bib-0040] Timmerman, V. , A. V. Strickland , and S. Zuchner . 2014 Genetics of Charcot‐Marie‐Tooth (CMT) disease within the frame of the human genome project success. Genes 5:13–32. 24705285 10.3390/genes5010013 PMC3978509

[brb3451-bib-0041] Verhoeven, K. , K. G. Claeys , S. Zuchner , J. M. Schroder , J. Weis , C. Ceuterick , et al. 2006 MFN2 mutation distribution and genotype/phenotype correlation in Charcot‐Marie‐Tooth type 2. Brain 129:2093–2102. 16714318 10.1093/brain/awl126

[brb3451-bib-0042] Watanabe, M. , N. Yamamoto , N. Ohkoshi , H. Nagata , Y. Kohno , A. Hayashi , et al. 2002 Corticosteroid‐ responsive asymmetric neuropathy with a myelin protein zero gene mutation. Neurology 59:767–769. 12221176 10.1212/wnl.59.5.767

[brb3451-bib-0043] Weis, J. , S. Brandner , M. Lammens , C. Sommer , and J. M. Vallat . 2012 Processing of nerve biopsies: a practical guide for neuropathologists. Clin. Neuropathol. 31:7–23. 22192700 10.5414/NP300468 PMC3663462

[brb3451-bib-0044] Zuchner, S. , I. V. Mersiyanova , M. Muglia , N. Bissar‐Tadmouri , J. Rochelle , E. L. Dadali , et al. 2004 Mutations in the mitochondrial GTPase mitofusin 2 cause Charcot‐Marie‐Tooth neuropathy type 2A. Nat. Genet. 36:449–451. 15064763 10.1038/ng1341

