# Supplementary material for: Underestimated associated features in CMT neuropathies: clinical indicators for the causative gene?
Source: Brain Behav. 2016 Mar 4;6(4):e00451. doi: 10.1002/brb3.451 (PMC4782242; doi:10.1002/brb3.451)
Supplement: Supplementary file 1 — Appendix S1. Additional symptoms and features in patients with CMT reported in the literature. [file BRB3-6-e00451-s001.pdf]

Appendix S1: Additional symptoms and features in patients with CMT reported in the literature.

Mutated gene	Severe sensory	Vocal cords	Tremor	UL pre-dominant	Contrac-tures	Scoliosis	Hand deformities	Skeletal	Deafness	Cognitive impairment	Bulbar
PMP22	-	-	+[1,2]	-	+[3]	+[1,2,4]	+[1,2]	cong. hip dysplasia, ulnar deviated hand, lordosis[5], chest deformity[6]	+ [1,2]	+[7]	+[3,4]
MPZ	+[8]	+[9]	+[10]	-	+[11]	+[12,8, 11]	+[8]	chest deformity[13]	+[12,14]	✚	+[15,11]
GJB1	-	+[19]	+[20]	-	-	+[12]	-	-	+[12]	+[21]	+[21]
PRX	-	-	+[46]	-	-	+[46,47]	+[48]	kyphosis[49]	+[47]	-	-
EGR2	-	+[25,26]	+[27]	-	+[28]	+[29,30]	+[31,30]	hip dysplasia[32]	+[25]	-	+[32,26]
PLEKHG5	-	-	-	-	-	-	-	spine deformity[83]	-	-	-
MFN2	-	+[16]	+[16]	-	+[16]	+[16]	-	hyperlordosis, kyphosis[17]	+[16]	+[18]	+[168]
GDAP1	-	+[51]	-	-	+[51]	+[52]	+[51]	joint laxity[52], kyphosis[53], chest deformity[54]	-	-	+[52]
PDK3	-	-	+[74]	-	-	-	-	-	+[74]	-	-
SURF1	-	-	-	-	-	+[90]	-	-	+[90]	-	-
DHTKD1	-	-	-	-	-	-	-	-	-	-	-
INF2	-	-	+[65]	-	-	+[65]	+[65]	partial syndactyly[66], **ulnar deviated hands**, **kyphosis**	+[65]	+[66]	-
KIF1B	-	-	-	-	-	-	-	lordosis[75]	-	-	-
DYNC1H1	-	-	+[77]	-	-	-	-	lordosis[77]	-	+[77]	-
LMNA	-	-	-	-	-	+[78]	+[78]	hyperlordosis[79]	-	-	-
NEFL	+[22]	-	+[22]	-	+[22]	+[23]	+[24]	lordosis[23]	+[22]	+[22]	+[22]
TRIM2	-	-	-	-	-	-	-	-	-	-	-
GARS	-	-	-	+[40]	-	+[41]	-	-	-	-	-
KARS	-	-	-	-	-	-	-	-	-	-	-
AARS	-	-	-	-	-	-	-	-	+[76]	-	-
MARS	-	-	-	-	-	-	-	-	-	-	-
HARS	-	-	-	-	-	-	-	-	-	-	-
YARS	-	-	-	-	-	-	-	-	-	-	-
HINT1	-	-	-	-	-	-	-	-	-	-	-
LRSAM1	-	-	-	-	-	-	-	-	+[82]	-	-
SH3TC2	-	-	+[43]	-	-	+[43]	+[44]	-	+[43]	-	+[45]
RAB7	+[50]	-	-	-	-	+[50]	-	-	-	-	-
FIG4	-	-	+[60]	-	-	+[60]	+[61]	-	-	-	-
DNM2	-	-	-	-	-	-	-	-	-	+[64]	-
MTMR2	-	+[67]	-	-	-	+[68]	+[67]	chest deformity[67]	-	-	+[2]
TFG	-	-	+[69]	-	-	-	-	-	+[69]	-	+[69]
LITAF	-	-	+[70]	✚	-	+[71]	-	-	+[71]	✚	-
FGD4	+[80]	-	+[81]	-	-	+[81]	-	-	-	-	-
SBF2	-	-	-	-	-	+[86]	+[86]	-	+[87]	-	-
NDRG1	-	-	+[55]	-	-	+[55]	+[56]	-	+[55]	-	+[57]
HSPB8	-	-	-	-	-	+[42]	-	-	-	-	-
HSPB1	-	-	-	-	-	-	+[91]	-	-	-	-
HSJ1	-	-	-	-	-	-	-	-	-	-	-
MED25	-	-	-	-	-	-	-	-	-	-	-
IFRD1	-	-	+[92]	-	-	-	-	-	+[92]	-	+[92]
TRPV4	-	+[33]	+[34]	-	+[35]	+[34]	-	short stature[36], lordosis[37], cong. hip dislocation[38]	+[39]	-	+[37]
SLC12A6	-	-	+[88]	-	-	-	-	kyphosis[89], joint laxity[88]	-	+[89]	-
HK1	-	+[58]	+[58]	-	-	+[59]	+[58]	-	-	+[59]	-
AIFM1	-	-	-	-	-	-	-	-	+[62]	+[62]	-
PRPS1	-	-	-	-	-	-	-	-	+[63]	-	-
FBLN5	-	-	-	-	-	+[72]	+[73]	joint laxity, lordosis[72]	-	-	-
GNB4	-	-	-	-	-	-	-	-	-	-	-
SBF1	-	-	-	-	-	+[84]	-	short stature, syndactyly and webbing[85]	-	+[85]	+[85]

Appendix S1 (continued): Additional symptoms and features in patients with CMT reported in the literature.

Mutated gene	Upper motor neuron	Fascicu-lations	Facial weak-ness	Early proximal weakness	Pain	Paresthesia	Early onset	Eye involvement	CTS	Respiratory
PMP22	-	+[5]	+[5,2]	-	+[5,3]	+[5]	+[5,3]	optic neuritis[93], pupill.[5,3]	**+**	diaphragmal weakness[5,2], **respiratory failure**, resp. distress at birth[3]
MPZ	-	**+**	+[94,95]	+[15,96]	+ [97,98]	+[97,98]	+[99,8, 11]	pupill.[12,98], ophthalmoparesis[94], optic atrophy[167]	**+**	resp. failure[97,100,95], chronic cough[101], resp. distress neonatal[15,11], resp. obstruction, stridor[9]
GJB1	+[21]	-	-	-	**+**	+[21]	+[20]	pupill.[102]	-	-
PRX	-	+(to)[47]	-	+[47]	-	+[48,109]	+[48]	glaucoma[110]	+[46]	restr. resp. failure[110]
EGR2	-	+[26]	+[26]	+[104]	-	-	+[26]	ophthalmoparesis [25,26], strabismus[25,32,105], pupill.[26]	-	restrictive pulmonary disease, resp. failure (with death)[26]
PLEKHG5	-	-	-	-	-	-	-	-	-	-
MFN2	+[16]	-	+[17]	+[16]	+[16]	**+**	+[17]	bilat. optic atrophy[16], ophthalmoparesis [18], pupill.[102]	-	resp. failure[103]
GDAP1	-	-	+[52]	-	+[112]	-	+[52]	optic atrophy[112]	-	diaphragmal weakness, resp. failure[52]
PDK3	-	-	-	-	-	-	+[74]	-	-	-
SURF1	-	-	-	-	-	-	-	-	-	-
DHTKD1	-	-	-	-	-	-	-	-	-	-
INF2	-	-	-	-	+[65]	-	+[65]	-	-	-
KIF1B	-	-	-	-	-	-	-	-	-	-
DYNC1H1	-	-	-	+[77]	+[77]	-	+[77]	-	-	-
LMNA	-	-	-	+[125]	-	-	-	-	-	-
NEFL	+[24]	+[23]	+[22]	-	-	**+**	+[22]	-	-	-
TRIM2	-	-	-	-	-	-	+[133]	-	-	-
GARS	-	-	-	-	-	-	-	-	-	-
KARS	-	-	-	-	-	-	+[127]	-	-	-
AARS	-	-	-	-	-	-	-	-	-	-
MARS	-	-	-	-	+[129]	-	-	-	-	-
HARS	-	-	-	-	+[131]	-	-	-	-	-
YARS	-	-	-	-	-	-	-	-	-	-
HINT1	-	+(ext)[132]	-	-	-	-	-	-	-	-
LRSAM1	-	+(ext)[128]	-	-	-	-	-	-	-	-
SH3TC2	-	+(to)[43]	+[106]	+[43]	+[44]	-	+[43]	pupill.[106]	-	resp. failure[107], left diaphragm paralysis[108]
RAB7	-	+(ext)[111]	-	-	+[50]	-	-	-	-	-
FIG4	-	-	+[60]	+[61]	+[60]	+[60]	+[60]	ophthalmoparesis [60]	+ [115]	death due to resp. failure[116], elevated hemi-diaphragm[60]
DNM2	-	-	+[107]	-	-	+[108]	+[119]	cataracts, ophthalmoparesis, strabismus[117]	-	-
MTMR2	-	-	+[67]	+[68]	-	-	+[120]	-	-	death due to resp. failure[68], chronic stridor[67]
TFG	+[121]	+(ext, tr, to)[69]	+[69]	+[69]	+[69]	+[122]	-	-	-	resp. failure[69]
LITAF	-	-	-	-	+[71]	+[123]	-	-	**+**	-
FGD4	-	-	-	-	-	-	+[126]	pupill.[80]	-	-
SBF2	-	-	-	-	+[86]	-	-	early-onset glaucoma[130]	-	-
NDRG1	-	-	+[113]	-	-	-	+[114]	pupill.[56]	-	-
HSPB8	-	-	-	-	-	-	-	-	-	-
HSPB1	-	+(ext)[91]	-	-	+[91]	-	-	-	-	-
HSJ1	-	-	-	-	-	+[134]	-	-	-	-
MED25	-	-	-	-	-	+[124]	-	-	-	-
IFRD1	+[92]	-	-	-	-	-	-	ophthalmoparesis[92]	-	-
TRPV4	-	-	+[33]	+[33]	-	-	+[33]	strabismus[36], ophthalmoparesis [33],pupill.[36]	+[36]	intercostal weakness, stridor, resp. failure[33]
SLC12A6	+[89]	-	+[89]	-	-	-	+[89]	strabismus[89]	-	-
HK1	-	-	+[58]	+[59]	-	-	-	-	-	-
AIFM1	-	+[62]	-	-	-	-	+[62]	-	-	-
PRPS1	-	-	-	-	-	-	-	optic atrophy bilat.[63]	-	-
FBLN5	-	-	-	-	-	+[73]	-	macular degeneration[72]	+[72]	resp. failure[72]
GNB4	-	-	-	-	-	-	-	-	-	-
SBF1	-	-	+[85]	-	-	-	-	strabismus, pupill.[85]	-	-

Appendix S1 (continued): Additional symptoms and features in patients with CMT reported in the literature.

Mutated gene	SAS	Autonomous involvement	Dys-mor-phic	CNS/ cranial nerves	Brain imaging	WM involve-ment (MRI)	Severe slow NCV	Hyper-trophic nerve roots	Hyper-trophic cranial nerves	Low blood cells	Additional
PMP22	+ [135]	ulcers[5], U+D urgency[136], **U**+D incontinence [6], **OH**, **hyperhidrosis**	+ [137]	nystagmus [5,3], abnormal eye pursuit[5]	reduced WM[1]	+[7]	+[138, 2,4]	+ [136,2]	-	-	RLS[135], calf hypertrophy[1], **cold induced hand cramps**
MPZ	+ [135]	ED, OH, U urgency[97], **U incontinence**, atonic bladder[101], **hyperhidrosis**	-	-	**plump lateral ventricles**	+ [139]	+[12, 15,11]	+[140, 141, 142]	+[143, 142]	-	RLS[135], calf hypertrophy[12], asymmetric weakness[144], **cold induced hand cramps**
GJB1	+ [135]	-	-	transient leukence-phalopathia[151], stroke-like episodes[152], ataxia[21], tremor, dysdiadochokinesia, saccadic pursuit, nystagmus, dysmetria,[148]	cerebellar/ spinal cord atrophy [148]	+[151]	-	-	-	-	RLS[135], asymmetric weakness[153]
PRX	-	-	+[49]	dysmetria, nystagmus hypodiadohokinesia, [46]	large cisterna magna[48]	-	+[46, 109]	-	-	-	cardiac insuficiency+ cardiomyopathy [47]
EGR2	-	-	-	nystagmus[105]	-	-	+[104, 105]	-	-	-	-
PLEKHG5	-	-	-	-	-	-	-	-	-	-	-
MFN2	-	ED, hyperhidrosis [145], **bladder urgency**	+ [146]	fatal subacute encephalopathy[147], dysmetria, dysdiado-chokinesia, saccadic pursuit, nystagmus[148]	spinal[149]/ cerebellar/ brain[150] atrophy, hyperin-tensities[16]	+[16]	-	-	-	-	scapular winging [103], **RLS**, **cold induced hand cramps**
GDAP1	+[52]	D incontinence D urgency[52], hyperhidrosis [54]	-	-	-	-	+[159]	-	-	-	-
PDK3	-	-	-	-	-	-	-	-	-	-	-
SURF1	-	-	-	nystagmus, cerebellar ataxia[90]	hyperintense lesions putamen+pe riaqueductal [90]	-	-	-	-	-	lactic acidosis under fasting conditions[90]
DHTKD1	-	-	-	-	-	-	-	-	-	-	-
INF2	-	-	-	-	enlarged ventricles [66]	+[65]	-	-	-	-	nephrotic syndrome/ proteinuria, FSGS ESRD[65]
KIF1B	-	-	-	-	-	-	-	-	-	-	-
DYNC1H1	-	-	-	-	-	-	-	-	-	-	proximal > distal weakness, periscapular atrophy + weakness[77]
LMNA	-	-	-	-	-	-	-	-	-	-	-
NEFL	-	chronic vomiting[22]	-	nystagmus, cerebellar signs[23]	enlarged ventricles, cerebellar atrophy[24]	-	-	-	-	-	hyperkeratosis [154], calf hypertrophy [155]
TRIM2	-	-	-	-	-	-	-	-	-	-	-
GARS	-	-	-	-	-	-	-	-	-	-	cold induced hand cramps[40]
KARS	-	-	+ [127]	-	-	-	-	-	-	-	self-abusive behavior, developmental delay[127]
AARS	-	-	-	-	-	-	-	-	-	-	-
MARS	-	-	-	-	-	-	-	-	-	-	proximal = distal weakness in LL[129]
HARS	-	-	-	-	-	-	-	-	-	-	-
YARS	-	-	-	-	-	-	-	-	-	-	-
HINT1	-	-	-	-	-	-	-	-	-	-	myokymia, neuromyotonia [132]
LRSAM1	-	U urgency, ED[128], mutilating arthropathy	-	-	-	-	-	-	-	-	-

		[166]									
SH3TC2	+ [107]	-	-	VII-X + XII cranial nerve involvement[45], tongue atrophy + weakness[106]	unilateral brain atrophy [106]	+[43]	+[107]	-	-	-	-
RAB7	-	ulcers, infections, amputations, hypohidrosis [157], pre-syncopes[158]	+ [157]	nystagmus[50]	cerebellar degeneration [50]	-	-	-	-	-	-
FIG4	+[60]	-	-	tongue weakness[60]	cerebellar/ brain atrophy[60]	-	+[60]	-	-	-	involuntary movements, asymmetric weakness[60]
DNM2	-	-	-	-	-	-	-	-	-	+[117]	-
MTMR2	+[2]	-	+[68]	facial synkinesia[68], tongue atrophy, masticatory weakness[2]	-	-	+[163]	-	-	-	acrocyanosis [164]
TFG	-	constipation [69]	-	-	myelin pallor in spinal cord columns [121]	-	-	-	-	-	hyperglycemia, hyperlipidemia [69], myotonia, proximal>distal weakness, scapular + pelvic girdle atrophy[122]
LITAF	-	**OH**	-	-	-	-	+[165]	-	-	-	**RLS**
FGD4	-	-	+ [126]	-	-	-	+[80]	-	-	-	-
SBF2	-	-	-	cranial nerve involvement[87]	-	-	-	-	-	-	-
NDRG1	-	ulcers[113], bowel dys-function[55]	-	nystagmus[160], tongue atrophy[113]	-	+[114]	+[161]	-	-	-	-
HSPB8	-	-	-	-	-	-	-	-	-	-	-
HSPB1	-	-	-	-	-	-	-	-	-	-	-
HSJ1	-	-	-	-	-	-	-	-	-	-	-
MED25	-	-	-	-	-	-	-	-	-	-	oedema[124]
IFRD1	-	-	-	coordination deficits, dysmetria, dysdiadochokinesia, nystagmus[92]	cerebellar atrophy[92]	-	-	-	-	-	proximal > distal weakness[92]
TRPV4	+[33]	U incontinence + urgency[39]	+[36]	abducens nerve palsy[156]	-	-	-	-	-	-	neck + trunk weakness[37], scapular winging[34]
SLC12A6	-	nocturnal vomiting[88]	+[89]	coordination deficits[88]	corpus callosum agenesis, enlarged ventricles, brain atrophy[89]	+[89]	-	-	-	-	epilepsy[89]
HK1	-	-	-	-	-	-	-	-	-	-	-
AIFM1	-	-	-	-	-	+[162]	-	-	-	-	-
PRPS1	-	-	-	-	-	-	-	-	-	-	-
FBLN5	-	chronic diarrhea[72]	+[72]	-	-	-	-	-	-	-	scapular winging, hyperelastic skin[72]
GNB4	-	-	-	-	-	-	-	-	-	-	-
SBF1	-	incontinence [85]	+[85]	-	brain atrophy[85]	-	-	-	-	-	-

Concerning PMP22: HNPP data are not included, cong. = congenital, CTS = carpal tunnel syndrome, pupill. = pupillary abnormality, resp. = respiratory, bilat. = bilateral, to = tongue, restr. = restrictive, ext = extremities, tr = trunk, SAS = sleep apnea syndrome, CNS = central nervous system, WM = white matter, MRI = magnetic resonance imaging, NCV = nerve conduction velocity, RLS = restless legs syndrome, ED = erectile dysfunction, OH = orthostatic hypotension, U = urinary, D = defecatory, FSGS = focal segmental glomerular sclerosis, ESRD = end stage renal disease, LL = lower limbs. Bold: new findings in current patient cohort; underlined: findings in current study that have been reported previously; not underlined and not bold: literature findings. Colors of the genes indicate their function within the peripheral nervous system. Yellow: myelination; light blue: mitochondrial; green: cytoskeletal stability and motor proteins; pink: RNA and protein metabolism; dark blue: protein folding; red: membrane traffic; grey: other/unknown. Violet: Transcription regulation. Brown: Channel/Transporter. Severe slow NCV < 10 m/s. Early onset < 18 month of age. Chest deformity = other than scoliosis.

References:

1. van Paassen BW, van der Kooi AJ, van Spaendonck-Zwarts KY, Verhamme C, Baas F, de Visser M (2014) PMP22 related neuropathies: Charcot-Marie-Tooth disease type 1A and Hereditary Neuropathy with liability to Pressure Palsies. Orphanet J Rare Dis 9:38.
2. Tyson J, Ellis D, Fairbrother U, King RH, Muntoni F, Jacobs J et al. (1997) Hereditary demyelinating neuropathy of infancy. A genetically complex syndrome. Brain 120 (Pt 1):47-63.
3. Hui-Chou HG, Hashemi SS, Hoke A, Dellon AL (2011) Clinical implications of peripheral myelin protein 22 for nerve compression and neural regeneration: a review. J Reconstr Microsurg 27 (1):67-74.
4. Simonati A, Fabrizi GM, Pasquinelli A, Taioli F, Cavallaro T, Morbin M et al. (1999) Congenital hypomyelination neuropathy with Ser72Leu substitution in PMP22. Neuromuscul Disord 9 (4):257-261.
5. Marques W, Jr., Freitas MR, Nascimento OJ, Oliveira AB, Calia L, Melo A et al. (2005) 17p duplicated Charcot-Marie-Tooth 1A: characteristics of a new population. J Neurol 252 (8):972-979.
6. Thomas PK, Marques W, Jr., Davis MB, Sweeney MG, King RH, Bradley JL et al. (1997) The phenotypic manifestations of chromosome 17p11.2 duplication. Brain 120 (Pt 3):465-478.
7. Chanson JB, Echaniz-Laguna A, Blanc F, Lacour A, Ballonzoli L, Kremer S et al. (2013) Central nervous system abnormalities in patients with PMP22 gene mutations: a prospective study. J Neurol Neurosurg Psychiatry 84 (4):392-397.
8. Warner LE, Hilz MJ, Appel SH, Killian JM, Kolodry EH, Karpati G et al. (1996) Clinical phenotypes of different MPZ (P0) mutations may include Charcot-Marie-Tooth type 1B, Dejerine-Sottas, and congenital hypomyelination. Neuron 17 (3):451-460.
9. Benson B, Sulica L, Guss J, Blitzer A (2010) Laryngeal neuropathy of Charcot-Marie-Tooth disease: further observations and novel mutations associated with vocal fold paresis. Laryngoscope 120 (2):291-296.
10. Choi BO, Kim SB, Kanwal S, Hyun YS, Park SW, Koo H et al. (2011) MPZ mutation in an early-onset Charcot-Marie-Tooth disease type 1B family by genome-wide linkage analysis. Int J Mol Med 28 (3):389-396.
11. Smit LS, Roofthooft D, van Ruissen F, Baas F, van Doorn PA (2008) Congenital hypomyelinating neuropathy, a long term follow-up study in an affected family. Neuromuscul Disord 18 (1):59-62.
12. Hattori N, Yamamoto M, Yoshihara T, Koike H, Nakagawa M, Yoshikawa H et al. (2003) Demyelinating and axonal features of Charcot-Marie-Tooth disease with mutations of myelin-related proteins (PMP22, MPZ and Cx32): a clinicopathological study of 205 Japanese patients. Brain 126 (Pt 1):134-151.
13. Kochanski A, Drac H, Kabzinska D, Ryniewicz B, Rowinska-Marcinska K, Nowakowski A et al. (2004) A novel MPZ gene mutation in congenital neuropathy with hypomyelination. Neurology 62 (11):2122-2123.
14. Gabreels-Festen A (2002) Dejerine-Sottas syndrome grown to maturity: overview of genetic and morphological heterogeneity and follow-up of 25 patients. J Anat 200 (4):341-356.
15. Tachi N, Kozuka N, Ohya K, Chiba S, Yamashita S (1998) A small direct tandem duplication of the myelin protein zero gene in a patient with Dejerine-Sottas disease phenotype. J Neurol Sci 156 (2):167-171.
16. Choi BO, Lee MS, Shin SH, Hwang JH, Choi KG, Kim WK et al. (2004) Mutational analysis of PMP22, MPZ, GJB1, EGR2 and NEFL in Korean Charcot-Marie-Tooth neuropathy patients. Hum Mutat 24 (2):185-186.
17. Polke JM, Laura M, Pareyson D, Taroni F, Milani M, Bergamin G et al. (2011) Recessive axonal Charcot-Marie-Tooth disease due to compound heterozygous mitofusin 2 mutations. Neurology 77 (2):168-173.
18. Casasnovas C, Banchs I, Cassereau J, Gueguen N, Chevrollier A, Martinez-Matos JA et al. (2010) Phenotypic spectrum of MFN2 mutations in the Spanish population. J Med Genet 47 (4):249-256.
19. Li QH, Liu KX, Feng JL, Zeng AY, Li H, Wu L et al. (2010) [A new mutation in the GJB1 gene of a Chinese family with Charcot-Marie-Tooth disease associated with vocal cord paresis]. Zhonghua Yi Xue Yi Chuan Xue Za Zhi 27 (5):497-500.
20. Yiu EM, Geevasinga N, Nicholson GA, Fagan ER, Ryan MM, Ouvrier RA (2011) A retrospective review of X-linked Charcot-Marie-Tooth disease in childhood. Neurology 76 (5):461-466.
21. Kleopa KA, Scherer SS (2006) Molecular genetics of X-linked Charcot-Marie-Tooth disease. NeuroMolecular Medicine 8 (1-2):107-122.
22. Jordanova A, Thomas FP, Guergueltcheva V, Tournev I, Gondim FA, Ishpekova B et al. (2003) Dominant intermediate Charcot-Marie-Tooth type C maps to chromosome 1p34-p35. Am J Hum Genet 73 (6):1423-1430.
23. Miltenberger-Miltenyi G, Janecke AR, Wanschitz JV, Timmerman V, Windpassinger C, Auer-Grumbach M et al. (2007) Clinical and electrophysiological features in Charcot-Marie-Tooth disease with mutations in the NEFL gene. Arch Neurol 64 (7):966-970.
24. Abe A, Numakura C, Saito K, Koide H, Oka N, Honma A et al. (2009) Neurofilament light chain polypeptide gene mutations in Charcot-Marie-Tooth disease: nonsense mutation probably causes a recessive phenotype. J Hum Genet 54 (2):94-97.
25. Pareyson D, Taroni F, Botti S, Morbin M, Baratta S, Lauria G et al. (2000) Cranial nerve involvement in CMT disease type 1 due to early growth response 2 gene mutation. Neurology 54 (8):1696-1698.
26. Szigeti K, Wiszniewski W, Saifi GM, Sherman DL, Sule N, Adesina AM et al. (2007) Functional, histopathologic and natural history study of neuropathy associated with EGR2 mutations. Neurogenetics 8 (4):257-262.
27. Yoshihara T, Kanda F, Yamamoto M, Ishihara H, Misu K, Hattori N et al. (2001) A novel missense mutation in the early growth response 2 gene associated with late-onset Charcot--Marie--Tooth disease type 1. J Neurol Sci 184 (2):149-153.
28. Funalot B, Topilko P, Arroyo MA, Sefiani A, Hedley-Whyte ET, Yoldi ME et al. (2012) Homozygous deletion of an EGR2 enhancer in congenital amyelinating neuropathy. Ann Neurol 71 (5):719-723.
29. Mikesova E, Huhne K, Rautenstrauss B, Mazanec R, Barankova L, Vyhnalek M et al. (2005) Novel EGR2 mutation R359Q is associated with CMT type 1 and progressive scoliosis. Neuromuscul Disord 15 (11):764-767.
30. Numakura C, Shirahata E, Yamashita S, Kanai M, Kijima K, Matsuki T et al. (2003) Screening of the early growth response 2 gene in Japanese patients with Charcot–Marie–Tooth disease type 1. Journal of the Neurological Sciences 210 (1-2):61-64.
31. Safka Brozkova D, Nevsimalova S, Mazanec R, Rautenstrauss B, Seeman P (2012) Charcot-Marie-Tooth neuropathy due to a novel EGR2 gene mutation with mild phenotype--usefulness of human mapping chip linkage analysis in a Czech family. Neuromuscul Disord 22 (8):742-746.
32. Boerkoel CF, Takashima H, Bacino CA, Daentl D, Lupski JR (2001) EGR2 mutation R359W causes a spectrum of Dejerine-Sottas neuropathy. Neurogenetics 3 (3):153-157.

33. McEntagart ME, Reid SL, Irrthum A, Douglas JB, Eyre KE, Donaghy MJ et al. (2005) Confirmation of a hereditary motor and sensory neuropathy IIC locus at chromosome 12q23-q24. Ann Neurol 57 (2):293-297.
34. Echaniz-Laguna A, Dubourg O, Carlier P, Carlier RY, Sabouraud P, Pereon Y et al. (2014) Phenotypic spectrum and incidence of TRPV4 mutations in patients with inherited axonal neuropathy. Neurology 82 (21):1919-1926.
35. Auer-Grumbach M, Olschewski A, Papic L, Kremer H, McEntagart ME, Uhrig S et al. (2010) Alterations in the ankyrin domain of TRPV4 cause congenital distal SMA, scapuloperoneal SMA and HMSN2C. Nat Genet 42 (2):160-164.
36. Chen DH, Sul Y, Weiss M, Hillel A, Lipe H, Wolff J et al. (2010) CMT2C with vocal cord paresis associated with short stature and mutations in the TRPV4 gene. Neurology 75 (22):1968-1975.
37. Dyck PJ, Litchy WJ, Minnerath S, Bird TD, Chance PF, Schaid DJ et al. (1994) Hereditary motor and sensory neuropathy with diaphragm and vocal cord paresis. Ann Neurol 35 (5):608-615.
38. Aharoni S, Harlalka G, Offiah A, Shuper A, Crosby AH, McEntagart M (2011) Striking phenotypic variability in familial TRPV4-axonal neuropathy spectrum disorder. Am J Med Genet A 155A (12):3153-3156.
39. Landoure G, Zdebik AA, Martinez TL, Burnett BG, Stanescu HC, Inada H et al. (2010) Mutations in TRPV4 cause Charcot-Marie-Tooth disease type 2C. Nat Genet 42 (2):170-174.
40. Sambuughin N, Sivakumar K, Selenge B, Lee HS, Friedlich D, Baasanjav D et al. (1998) Autosomal dominant distal spinal muscular atrophy type V (dSMA-V) and Charcot-Marie-Tooth disease type 2D (CMT2D) segregate within a single large kindred and map to a refined region on chromosome 7p15. J Neurol Sci 161 (1):23-28.
41. Ionasescu V, Searby C, Sheffield VC, Roklina T, Nishimura D, Ionasescu R (1996) Autosomal dominant Charcot-Marie-Tooth axonal neuropathy mapped on chromosome 7p (CMT2D). Hum Mol Genet 5 (9):1373-1375.
42. Nakhro K, Park JM, Kim YJ, Yoon BR, Yoo JH, Koo H et al. (2013) A novel Lys141Thr mutation in small heat shock protein 22 (HSPB8) gene in Charcot-Marie-Tooth disease type 2L. Neuromuscul Disord 23 (8):656-663.
43. Colomer J, Gooding R, Angelicheva D, King RH, Guillen-Navarro E, Parman Y et al. (2006) Clinical spectrum of CMT4C disease in patients homozygous for the p.Arg1109X mutation in SH3TC2. Neuromuscul Disord 16 (7):449-453.
44. Iguchi M, Hashiguchi A, Ito E, Toda K, Urano M, Shimizu Y et al. (2013) Charcot-Marie-Tooth disease type 4C in Japan: report of a case. Muscle Nerve 47 (2):283-286.
45. Yger M, Stojkovic T, Tardieu S, Maisonobe T, Brice A, Echaniz-Laguna A et al. (2012) Characteristics of clinical and electrophysiological pattern of Charcot-Marie-Tooth 4C. J Peripher Nerv Syst 17 (1):112-122.
46. Marchesi C, Milani M, Morbin M, Cesani M, Lauria G, Scaioli V et al. (2010) Four novel cases of periaxin-related neuropathy and review of the literature. Neurology 75 (20):1830-1838.
47. Takashima H, Boerkoel CF, De Jonghe P, Ceuterick C, Martin JJ, Voit T et al. (2002) Periaxin mutations cause a broad spectrum of demyelinating neuropathies. Ann Neurol 51 (6):709-715.
48. Delague V, Bareil C, Tuffery S, Bouvagnet P, Chouery E, Koussa S et al. (2000) Mapping of a new locus for autosomal recessive demyelinating Charcot-Marie-Tooth disease to 19q13.1-13.3 in a large consanguineous Lebanese family: exclusion of MAG as a candidate gene. Am J Hum Genet 67 (1):236-243.
49. Auer-Grumbach M, Fischer C, Papic L, John E, Plecko B, Bittner RE et al. (2008) Two novel mutations in the GDAP1 and PRX genes in early onset Charcot-Marie-Tooth syndrome. Neuropediatrics 39 (1):33-38.
50. Houlden H, King RH, Muddle JR, Warner TT, Reilly MM, Orrell RW et al. (2004) A novel RAB7 mutation associated with ulcero-mutilating neuropathy. Ann Neurol 56 (4):586-590.
51. Azzedine H, Ruberg M, Ente D, Gilardeau C, Perie S, Wechsler B et al. (2003) Variability of disease progression in a family with autosomal recessive CMT associated with a S194X and new R310Q mutation in the GDAP1 gene. Neuromuscul Disord 13 (4):341-346.
52. Sevilla T, Jaijo T, Nauffal D, Collado D, Chumillas MJ, Vilchez JJ et al. (2008) Vocal cord paresis and diaphragmatic dysfunction are severe and frequent symptoms of GDAP1-associated neuropathy. Brain 131 (Pt 11):3051-3061.
53. Baxter RV, Ben Othmane K, Rochelle JM, Stajich JE, Hulette C, Dew-Knight S et al. (2002) Ganglioside-induced differentiation-associated protein-1 is mutant in Charcot-Marie-Tooth disease type 4A/8q21. Nat Genet 30 (1):21-22.
54. Kabzinska D, Niemann A, Drac H, Huber N, Potulska-Chromik A, Hausmanowa-Petrusewicz I et al. (2011) A new missense GDAP1 mutation disturbing targeting to the mitochondrial membrane causes a severe form of AR-CMT2C disease. Neurogenetics 12 (2):145-153.
55. Hunter M, Bernard R, Freitas E, Boyer A, Morar B, Martins IJ et al. (2003) Mutation screening of the N-myc downstream-regulated gene 1 (NDRG1) in patients with Charcot-Marie-Tooth Disease. Hum Mutat 22 (2):129-135.
56. Kalaydjieva L, Nikolova A, Turnev I, Petrova J, Hristova A, Ishpekova B et al. (1998) Hereditary motor and sensory neuropathy--Lom, a novel demyelinating neuropathy associated with deafness in gypsies. Clinical, electrophysiological and nerve biopsy findings. Brain 121 (Pt 3):399-408.
57. Dackovic J, Keckarevic-Markovic M, Komazec Z, Rakocevic-Stojanovic V, Lavrnic D, Stevic Z et al. (2008) Hereditary motor and sensory neuropathy Lom type in a Serbian family. Acta Myol 27:59-62.
58. Thomas PK, Kalaydjieva L, Youl B, Rogers T, Angelicheva D, King RHM et al. (2001) Hereditary motor and sensory neuropathy-russe: New autosomal recessive neuropathy in balkan gypsies. Annals of Neurology 50 (4):452-457.
59. Sevilla T, Martinez-Rubio D, Marquez C, Paradas C, Colomer J, Jaijo T et al. (2013) Genetics of the Charcot-Marie-Tooth disease in the Spanish Gypsy population: the hereditary motor and sensory neuropathy-Russe in depth. Clin Genet 83 (6):565-570.
60. Nicholson G, Lenk GM, Reddel SW, Grant AE, Towne CF, Ferguson CJ et al. (2011) Distinctive genetic and clinical features of CMT4J: a severe neuropathy caused by mutations in the PI(3,5)P(2) phosphatase FIG4. Brain 134 (Pt 7):1959-1971.
61. Cottenie E, Menezes MP, Rossor AM, Morrow JM, Yousry TA, Dick DJ et al. (2013) Rapidly progressive asymmetrical weakness in Charcot-Marie-Tooth disease type 4J resembles chronic inflammatory demyelinating polyneuropathy. Neuromuscul Disord 23 (5):399-403.
62. Cowchock FS, Duckett SW, Streletz LJ, Graziani LJ, Jackson LG (1985) X-linked motor-sensory neuropathy type-II with deafness and mental retardation: a new disorder. Am J Med Genet 20 (2):307-315.
63. Kim HJ, Sohn KM, Shy ME, Krajewski KM, Hwang M, Park JH et al. (2007) Mutations in PRPS1, which encodes the phosphoribosyl pyrophosphate synthetase enzyme critical for nucleotide biosynthesis, cause hereditary peripheral neuropathy with hearing loss and optic neuropathy (cmtx5). Am J Hum Genet 81 (3):552-558.
64. Saint-Lezer A, Sole G, Ribeiro E, Latour P, Mercie P, Longy-Boursier M (2012) [Non-fortuitous dynamin II mutation-related association: neutropenia and Charcot-Marie-Tooth disease]. Rev Neurol (Paris) 168 (4):367-370.

65. Boyer O, Nevo F, Plaisier E, Funalot B, Gribouval O, Benoit G et al. (2011) INF2 mutations in Charcot-Marie-Tooth disease with glomerulopathy. N Engl J Med 365 (25):2377-2388.
66. Mademan I, Deconinck T, Dinopoulos A, Voit T, Schara U, Devriendt K et al. (2013) De novo INF2 mutations expand the genetic spectrum of hereditary neuropathy with glomerulopathy. Neurology 81 (22):1953-1958.
67. Nouioua S, Hamadouche T, Funalot B, Bernard R, Bellatache N, Bouderba R et al. (2011) Novel mutations in the PRX and the MTMR2 genes are responsible for unusual Charcot-Marie-Tooth disease phenotypes. Neuromuscul Disord 21 (8):543-550.
68. Quattrone A, Gambardella A, Bono F, Aguglia U, Bolino A, Bruni AC et al. (1996) Autosomal recessive hereditary motor and sensory neuropathy with focally folded myelin sheaths: clinical, electrophysiologic, and genetic aspects of a large family. Neurology 46 (5):1318-1324.
69. Takashima H, Nakagawa M, Nakahara K, Suehara M, Matsuzaki T, Higuchi I et al. (1997) A new type of hereditary motor and sensory neuropathy linked to chromosome 3. Ann Neurol 41 (6):771-780.
70. Bennett CL, Shirk AJ, Huynh HM, Street VA, Nelis E, Van Maldergem L et al. (2004) SIMPLE mutation in demyelinating neuropathy and distribution in sciatic nerve. Ann Neurol 55 (5):713-720.
71. Saifi GM, Szigeti K, Wiszniewski W, Shy ME, Krajewski K, Hausmanowa-Petrusewicz I et al. (2005) SIMPLE mutations in Charcot-Marie-Tooth disease and the potential role of its protein product in protein degradation. Hum Mutat 25 (4):372-383.
72. Auer-Grumbach M, Weger M, Fink-Puches R, Papic L, Frohlich E, Auer-Grumbach P et al. (2011) Fibulin-5 mutations link inherited neuropathies, age-related macular degeneration and hyperelastic skin. Brain 134 (Pt 6):1839-1852.
73. Safka Brozkova D, Lassuthova P, Neupauerova J, Krutova M, Haberlova J, Stejskal D et al. (2013) Czech family confirms the link between FBLN5 and Charcot-Marie-Tooth type 1 neuropathy. Brain 136 (Pt 7):e232.
74. Kennerson ML, Yiu EM, Chuang DT, Kidambi A, Tso SC, Ly C et al. (2013) A new locus for X-linked dominant Charcot-Marie-Tooth disease (CMTX6) is caused by mutations in the pyruvate dehydrogenase kinase isoenzyme 3 (PDK3) gene. Hum Mol Genet 22 (7):1404-1416.
75. Saito M, Hayashi Y, Suzuki T, Tanaka H, Hozumi I, Tsuji S (1997) Linkage mapping of the gene for Charcot-Marie-Tooth disease type 2 to chromosome 1p (CMT2A) and the clinical features of CMT2A. Neurology 49 (6):1630-1635.
76. McLaughlin HM, Sakaguchi R, Giblin W, Program NCS, Wilson TE, Biesecker L et al. (2012) A recurrent loss-of-function alanyl-tRNA synthetase (AARS) mutation in patients with Charcot-Marie-Tooth disease type 2N (CMT2N). Hum Mutat 33 (1):244-253.
77. Weedon MN, Hastings R, Caswell R, Xie W, Paszkiewicz K, Antoniadi T et al. (2011) Exome sequencing identifies a DYNC1H1 mutation in a large pedigree with dominant axonal Charcot-Marie-Tooth disease. Am J Hum Genet 89 (2):308-312.
78. Bouhouche A, Birouk N, Azzedine H, Benomar A, Durosier G, Ente D et al. (2007) Autosomal recessive axonal Charcot-Marie-Tooth disease (ARCMT2): phenotype-genotype correlations in 13 Moroccan families. Brain 130 (Pt 4):1062-1075.
79. Tazir M, Azzedine H, Assami S, Sindou P, Nouioua S, Zemmouri R et al. (2004) Phenotypic variability in autosomal recessive axonal Charcot-Marie-Tooth disease due to the R298C mutation in lamin A/C. Brain 127 (Pt 1):154-163.
80. Houlden H, Hammans S, Katifi H, Reilly MM (2009) A novel Frabin (FGD4) nonsense mutation p.R275X associated with phenotypic variability in CMT4H. Neurology 72 (7):617-620.
81. Fabrizi GM, Taioli F, Cavallaro T, Ferrari S, Bertolasi L, Casarotto M et al. (2009) Further evidence that mutations in FGD4/frabin cause Charcot-Marie-Tooth disease type 4H. Neurology 72 (13):1160-1164.
82. Weterman MA, Sorrentino V, Kasher PR, Jakobs ME, van Engelen BG, Fluiter K et al. (2012) A frameshift mutation in LRSAM1 is responsible for a dominant hereditary polyneuropathy. Hum Mol Genet 21 (2):358-370.
83. Azzedine H, Zavadakova P, Plante-Bordeneuve V, Vaz Pato M, Pinto N, Bartesaghi L et al. (2013) PLEKHG5 deficiency leads to an intermediate form of autosomal-recessive Charcot-Marie-Tooth disease. Hum Mol Genet 22 (20):4224-4232.
84. Nakhro K, Park JM, Hong YB, Park JH, Nam SH, Yoon BR et al. (2013) SET binding factor 1 (SBF1) mutation causes Charcot-Marie-Tooth disease type 4B3. Neurology 81 (2):165-173.
85. Bohlega S, Alazami AM, Cupler E, Al-Hindi H, Ibrahim E, Alkuraya FS (2011) A novel syndromic form of sensory-motor polyneuropathy is linked to chromosome 22q13.31-q13.33. Clin Genet 79 (2):193-195.
86. Othmane KB, Johnson E, Menold M, Graham FL, Hamida MB, Hasegawa O et al. (1999) Identification of a new locus for autosomal recessive Charcot-Marie-Tooth disease with focally folded myelin on chromosome 11p15. Genomics 62 (3):344-349.
87. Gambardella A, Bolino A, Muglia M, Valentino P, Bono F, Oliveri RL et al. (1998) Genetic heterogeneity in autosomal recessive hereditary motor and sensory neuropathy with focally folded myelin sheaths (CMT4B). Neurology 50 (3):799-801.
88. Rudnik-Schoneborn S, Hehr U, von Kalle T, Bornemann A, Winkler J, Zerres K (2009) Andermann syndrome can be a phenocopy of hereditary motor and sensory neuropathy--report of a discordant sibship with a compound heterozygous mutation of the KCC3 gene. Neuropediatrics 40 (3):129-133.
89. Uyanik G, Elcioglu N, Penzien J, Gross C, Yilmaz Y, Olmez A et al. (2006) Novel truncating and missense mutations of the KCC3 gene associated with Andermann syndrome. Neurology 66 (7):1044-1048.
90. Echaniz-Laguna A, Ghezzi D, Chassagne M, Mayencon M, Padet S, Melchionda L et al. (2013) SURF1 deficiency causes demyelinating Charcot-Marie-Tooth disease. Neurology 81 (17):1523-1530.
91. Tang B, Liu X, Zhao G, Luo W, Xia K, Pan Q et al. (2005) Mutation analysis of the small heat shock protein 27 gene in chinese patients with Charcot-Marie-Tooth disease. Arch Neurol 62 (8):1201-1207.
92. Brkanac Z, Spencer D, Shendure J, Robertson PD, Matsushita M, Vu T et al. (2009) IFRD1 is a candidate gene for SMNA on chromosome 7q22-q23. Am J Hum Genet 84 (5):692-697.
93. Wakerley BR, Harman FE, Altmann DM, Malik O (2011) Charcot-Marie-Tooth disease associated with recurrent optic neuritis. J Clin Neurosci 18 (10):1422-1423.
94. Fabrizi GM, Cavallaro T, Morbin M, Simonati A, Taioli F, Rizzuto N (1999) Novel mutation of the P0 extracellular domain causes a Dejerine-Sottas syndrome. J Neurol Neurosurg Psychiatry 66 (3):386-389.
95. Szigeti K, Saifi GM, Armstrong D, Belmont JW, Miller G, Lupski JR (2003) Disturbance of muscle fiber differentiation in congenital hypomyelinating neuropathy caused by a novel myelin protein zero mutation. Ann Neurol 54 (3):398-402.
96. McMillan HJ, Santagata S, Shapiro F, Batish SD, Couchon L, Donnelly S et al. (2010) Novel MPZ mutations and congenital hypomyelinating neuropathy. Neuromuscul Disord 20 (11):725-729.
97. Stojkovic T, de Seze J, Dubourg O, Arne-Bes MC, Tardieu S, Hache JC et al. (2003) Autonomic and respiratory dysfunction in Charcot–Marie–Tooth disease due to Thr124Met mutation in the myelin protein zero gene. Clinical Neurophysiology 114 (9):1609-1614.

98. Floroskufi P, Panas M, Karadima G, Vassilopoulos D (2007) New mutation of the MPZ gene in a family with the Dejerine-Sottas disease phenotype. Muscle Nerve 35 (5):667-669.
99. Shy ME, Jani A, Krajewski K, Grandis M, Lewis RA, Li J et al. (2004) Phenotypic clustering in MPZ mutations. Brain 127 (Pt 2):371-384.
100. Taioli F, Cabrini I, Cavallaro T, Simonati A, Testi S, Fabrizi GM (2011) Dejerine-Sottas syndrome with a silent nucleotide change of myelin protein zero gene. J Peripher Nerv Syst 16 (1):59-64.
101. Nakamura N, Kawamura N, Tateishi T, Doi H, Ohyagi Y, Kira J (2009) [Predominant parasympathetic involvement in a patient with Charcot-Marie-Tooth disease caused by the MPZ Thr124Met mutation]. Rinsho Shinkeigaku 49 (9):582-585.
102. Houlden H, Reilly MM, Smith S (2009) Pupil abnormalities in 131 cases of genetically defined inherited peripheral neuropathy. Eye (Lond) 23 (4):966-974.
103. Zuchner S, De Jonghe P, Jordanova A, Claeys KG, Guergueltcheva V, Cherninkova S et al. (2006) Axonal neuropathy with optic atrophy is caused by mutations in mitofusin 2. Ann Neurol 59 (2):276-281.
104. Timmerman V, De Jonghe P, Ceuterick C, De Vriendt E, Lofgren A, Nelis E et al. (1999) Novel missense mutation in the early growth response 2 gene associated with Dejerine-Sottas syndrome phenotype. Neurology 52 (9):1827-1827.
105. Vandenberghe N, Upadhyaya M, Gatignol A, Boutrand L, Boucherat M, Chazot G et al. (2002) Frequency of mutations in the early growth response 2 gene associated with peripheral demyelinating neuropathies. J Med Genet 39 (12):e81.
106. Houlden H, Laura M, Ginsberg L, Jungbluth H, Robb SA, Blake J et al. (2009) The phenotype of Charcot-Marie-Tooth disease type 4C due to SH3TC2 mutations and possible predisposition to an inflammatory neuropathy. Neuromuscul Disord 19 (4):264-269.
107. Senderek J, Bergmann C, Stendel C, Kirfel J, Verpoorten N, De Jonghe P et al. (2003) Mutations in a gene encoding a novel SH3/TPR domain protein cause autosomal recessive Charcot-Marie-Tooth type 4C neuropathy. Am J Hum Genet 73 (5):1106-1119.
108. Aboussouan LS, Lewis RA, Shy ME (2007) Disorders of pulmonary function, sleep, and the upper airway in Charcot-Marie-Tooth disease. Lung 185 (1):1-7.
109. Boerkoel CF, Takashima H, Stankiewicz P, Garcia CA, Leber SM, Rhee-Morris L et al. (2001) Periaxin mutations cause recessive Dejerine-Sottas neuropathy. Am J Hum Genet 68 (2):325-333.
110. Renouil M, Stojkovic T, Jacquemont ML, Lauret K, Boue P, Fourmaintraux A et al. (2013) [Charcot-Marie-Tooth disease associated with periaxin mutations (CMT4F): Clinical, electrophysiological and genetic analysis of 24 patients]. Rev Neurol (Paris) 169 (8-9):603-612.
111. Meggouh F, Bienfait HM, Weterman MA, de Visser M, Baas F (2006) Charcot-Marie-Tooth disease due to a de novo mutation of the RAB7 gene. Neurology 67 (8):1476-1478.
112. Claramunt R, Pedrola L, Sevilla T, Lopez de Munain A, Berciano J, Cuesta A et al. (2005) Genetics of Charcot-Marie-Tooth disease type 4A: mutations, inheritance, phenotypic variability, and founder effect. J Med Genet 42 (4):358-365.
113. Butinar D, Zidar J, Leonardis L, Popovic M, Kalaydjieva L, Angelicheva D et al. (1999) Hereditary auditory, vestibular, motor, and sensory neuropathy in a Slovenian Roma (Gypsy) kindred. Ann Neurol 46 (1):36-44.
114. Echaniz-Laguna A, Degos B, Bonnet C, Latour P, Hamadouche T, Levy N et al. (2007) NDRG1-linked Charcot-Marie-Tooth disease (CMT4D) with central nervous system involvement. Neuromuscul Disord 17 (2):163-168.
115. Menezes MP, Waddell L, Lenk GM, Kaur S, MacArthur DG, Meisler MH et al. (2014) Whole exome sequencing identifies three recessive FIG4 mutations in an apparently dominant pedigree with Charcot-Marie-Tooth disease. Neuromuscul Disord 24 (8):666-670.
116. Zhang X, Chow CY, Sahenk Z, Shy ME, Meisler MH, Li J (2008) Mutation of FIG4 causes a rapidly progressive, asymmetric neuronal degeneration. Brain 131 (Pt 8):1990-2001.
117. Bitoun M, Stojkovic T, Prudhon B, Maurage CA, Latour P, Vermersch P et al. (2008) A novel mutation in the dynamin 2 gene in a Charcot-Marie-Tooth type 2 patient: clinical and pathological findings. Neuromuscul Disord 18 (4):334-338.
118. Fabrizi GM, Ferrarini M, Cavallaro T, Cabrini I, Cerini R, Bertolasi L et al. (2007) Two novel mutations in dynamin-2 cause axonal Charcot-Marie-Tooth disease. Neurology 69 (3):291-295.
119. Claeys KG, Zuchner S, Kennerson M, Berciano J, Garcia A, Verhoeven K et al. (2009) Phenotypic spectrum of dynamin 2 mutations in Charcot-Marie-Tooth neuropathy. Brain 132 (Pt 7):1741-1752.
120. Verny C, Ravise N, Leutenegger AL, Pouplard F, Dubourg O, Tardieu S et al. (2004) Coincidence of two genetic forms of Charcot-Marie-Tooth disease in a single family. Neurology 63 (8):1527-1529.
121. Fujita K, Yoshida M, Sako W, Maeda K, Hashizume Y, Goto S et al. (2011) Brainstem and spinal cord motor neuron involvement with optineurin inclusions in proximal-dominant hereditary motor and sensory neuropathy. J Neurol Neurosurg Psychiatry 82 (12):1402-1403.
122. Patroclo CB, Lino AM, Marchiori PE, Brotto MW, Hirata MT (2009) Autosomal dominant HMSN with proximal involvement: new Brazilian cases. Arq Neuropsiquiatr 67 (3b):892-896.
123. Gerding WM, Koetting J, Epplen JT, Neusch C (2009) Hereditary motor and sensory neuropathy caused by a novel mutation in LITAF. Neuromuscul Disord 19 (10):701-703.
124. Berghoff C, Berghoff M, Leal A, Morera B, Barrantes R, Reis A et al. (2004) Clinical and electrophysiological characteristics of autosomal recessive axonal Charcot-Marie-Tooth disease (ARCMT2B) that maps to chromosome 19q13.3. Neuromuscul Disord 14 (5):301-306.
125. Chaouch M, Allal Y, De Sandre-Giovannoli A, Vallat JM, Amer-el-Khedoud A, Kassouri N et al. (2003) The phenotypic manifestations of autosomal recessive axonal Charcot-Marie-Tooth due to a mutation in Lamin A/C gene. Neuromuscul Disord 13 (1):60-67.
126. De Sandre-Giovannoli A, Delague V, Hamadouche T, Chaouch M, Krahn M, Boccaccio I et al. (2005) Homozygosity mapping of autosomal recessive demyelinating Charcot-Marie-Tooth neuropathy (CMT4H) to a novel locus on chromosome 12p11.21-q13.11. J Med Genet 42 (3):260-265.
127. McLaughlin HM, Sakaguchi R, Liu C, Igarashi T, Pehlivan D, Chu K et al. (2010) Compound heterozygosity for loss-of-function lysyl-tRNA synthetase mutations in a patient with peripheral neuropathy. Am J Hum Genet 87 (4):560-566.
128. Guernsey DL, Jiang H, Bedard K, Evans SC, Ferguson M, Matsuoka M et al. (2010) Mutation in the gene encoding ubiquitin ligase LRSAM1 in patients with Charcot-Marie-Tooth disease. PLoS Genet 6 (8).
129. Gonzalez M, McLaughlin H, Houlden H, Guo M, Yo-Tsen L, Hadjivassilious M et al. (2013) Exome sequencing identifies a significant variant in methionyl-tRNA synthetase (MARS) in a family with late-onset CMT2. J Neurol Neurosurg Psychiatry 84 (11):1247-1249.

130. Azzedine H, Bolino A, Taieb T, Birouk N, Di Duca M, Bouhouche A et al. (2003) Mutations in MTMR13, a new pseudophosphatase homologue of MTMR2 and Sbf1, in two families with an autosomal recessive demyelinating form of Charcot-Marie-Tooth disease associated with early-onset glaucoma. Am J Hum Genet 72 (5):1141-1153.
131. Vester A, Velez-Ruiz G, McLaughlin HM, Program NCS, Lupski JR, Talbot K et al. (2013) A loss-of-function variant in the human histidyl-tRNA synthetase (HARS) gene is neurotoxic in vivo. Hum Mutat 34 (1):191-199.
132. Hahn AF, Parkes AW, Bolton CF, Stewart SA (1991) Neuromyotonia in hereditary motor neuropathy. J Neurol Neurosurg Psychiatry 54 (3):230-235.
133. Ylikallio E, Poyhonen R, Zimon M, De Vriendt E, Hilander T, Paetau A et al. (2013) Deficiency of the E3 ubiquitin ligase TRIM2 in early-onset axonal neuropathy. Hum Mol Genet 22 (15):2975-2983.
134. Gess B, Auer-Grumbach M, Schirmacher A, Strom T, Zitzelsberger M, Rudnik-Schoneborn S et al. (2014) HSJ1-related hereditary neuropathies: novel mutations and extended clinical spectrum. Neurology 83 (19):1726-1732.
135. Boentert M, Knop K, Schuhmacher C, Gess B, Okegwo A, Young P (2014) Sleep disorders in Charcot-Marie-Tooth disease type 1. J Neurol Neurosurg Psychiatry 85 (3):319-325.
136. Butefisch C, Gutmann L, Gutmann L (1999) Compression of spinal cord and cauda equina in Charcot-Marie-Tooth disease type 1A. Neurology 52 (4):890-891.
137. Moog U, Engelen JJ, Weber BW, Van Gelderen M, Steyaert J, Baas F et al. (2004) Hereditary motor and sensory neuropathy (HMSN) IA, developmental delay and autism related disorder in a boy with duplication (17)(p11.2p12). Genet Couns 15 (1):73-80.
138. Birouk N, Gouider R, Le Guern E, Gugenheim M, Tardieu S, Maisonobe T et al. (1997) Charcot-Marie-Tooth disease type 1A with 17p11.2 duplication. Clinical and electrophysiological phenotype study and factors influencing disease severity in 119 cases. Brain 120 (Pt 5):813-823.
139. Speevak MD, Farrell SA (2013) Charcot-Marie-Tooth 1B caused by expansion of a familial myelin protein zero (MPZ) gene duplication. Eur J Med Genet 56 (10):566-569.
140. Marchini C, Marsala SZ, Bendini M, Taioli F, Damante G, Lonigro IR et al. (2009) Myelin protein zero Val102fs mutation manifesting with isolated spinal root hypertrophy. Neuromuscul Disord 19 (12):849-852.
141. Kleopa KA, Sutton LN, Ong J, Tennekoon G, Telfeian AE (2002) Conus medulla-cauda compression from nerve root hypertrophy in a child with Dejerine-Sottas syndrome: improvement with laminectomy and duraplasty. Case report. J Neurosurg 97 (2 Suppl):244-247.
142. Simonati A, Fabrizi GM, Taioli F, Polo A, Cerini R, Rizzuto N (2002) Dejerine-Sottas neuropathy with multiple nerve roots enlargement and hypomyelination associated with a missense mutation of the transmembrane domain of MPZ/P0. J Neurol 249 (9):1298-1302.
143. Shizuka M, Ikeda Y, Watanabe M, Okamoto K, Shoji M, Ikegami T et al. (1999) A novel mutation of the myelin P(o) gene segregating Charcot-Marie-Toothdisease type 1B manifesting as trigeminal nerve thickening. J Neurol Neurosurg Psychiatry 67 (2):250-251.
144. Hayasaka K, Ohnishi A, Takada G, Fukushima Y, Murai Y (1993) Mutation of the myelin P0 gene in Charcot-Marie-tooth neuropathy type 1. Biochem Biophys Res Commun 194 (3):1317-1322.
145. Martikainen MH, Kytovuori L, Majamaa K (2014) Novel mitofusin 2 splice-site mutation causes Charcot-Marie-Tooth disease type 2 with prominent sensory dysfunction. Neuromuscul Disord 24 (4):360-364.
146. Chung KW, Suh BC, Cho SY, Choi SK, Kang SH, Yoo JH et al. (2010) Early-onset Charcot-Marie-Tooth patients with mitofusin 2 mutations and brain involvement. J Neurol Neurosurg Psychiatry 81 (11):1203-1206.
147. Boaretto F, Vettori A, Casarin A, Vazza G, Muglia M, Rossetto MG et al. (2010) Severe CMT type 2 with fatal encephalopathy associated with a novel MFN2 splicing mutation. Neurology 74 (23):1919-1921.
148. Caramins M, Colebatch JG, Bainbridge MN, Scherer SS, Abrams CK, Hackett EL et al. (2013) Exome sequencing identification of a GJB1 missense mutation in a kindred with X-linked spinocerebellar ataxia (SCA-X1). Hum Mol Genet 22 (21):4329-4338.
149. Del Bo R, Moggio M, Rango M, Bonato S, D'Angelo MG, Ghezzi S et al. (2008) Mutated mitofusin 2 presents with intrafamilial variability and brain mitochondrial dysfunction. Neurology 71 (24):1959-1966.
150. Genari AB, Borghetti VH, Gouvea SP, Bueno KC, dos Santos PL, dos Santos AC et al. (2011) Characterizing the phenotypic manifestations of MFN2 R104W mutation in Charcot-Marie-Tooth type 2. Neuromuscul Disord 21 (6):428-432.
151. Hanemann CO, Bergmann C, Senderek J, Zerres K, Sperfeld AD (2003) Transient, recurrent, white matter lesions in X-linked Charcot-Marie-Tooth disease with novel connexin 32 mutation. Arch Neurol 60 (4):605-609.
152. Anand G, Maheshwari N, Roberts D, Padeniya A, Hamilton-Ayers M, van der Knaap M et al. (2010) X-linked hereditary motor sensory neuropathy (type 1) presenting with a stroke-like episode. Dev Med Child Neurol 52 (7):677-679.
153. Tabaraud F, Lagrange E, Sindou P, Vandenberghe A, Levy N, Vallat JM (1999) Demyelinating X-linked Charcot-Marie-Tooth disease: unusual electrophysiological findings. Muscle Nerve 22 (10):1442-1447.
154. Mersiyanova IV, Perepelov AV, Polyakov AV, Sitnikov VF, Dadali EL, Oparin RB et al. (2000) A new variant of Charcot-Marie-Tooth disease type 2 is probably the result of a mutation in the neurofilament-light gene. Am J Hum Genet 67 (1):37-46.
155. Fabrizi GM, Cavallaro T, Angiari C, Bertolasi L, Cabrini I, Ferrarini M et al. (2004) Giant axon and neurofilament accumulation in Charcot-Marie-Tooth disease type 2E. Neurology 62 (8):1429-1431.
156. Donaghy M, Kennett R (1999) Varying occurrence of vocal cord paralysis in a family with autosomal dominant hereditary motor and sensory neuropathy. J Neurol 246 (7):552-555.
157. Auer-Grumbach M, De Jonghe P, Wagner K, Verhoeven K, Hartung HP, Timmerman V (2000) Phenotype-genotype correlations in a CMT2B family with refined 3q13-q22 locus. Neurology 55 (10):1552-1557.
158. Manganelli F, Pisciotta C, Provitera V, Taioli F, Iodice R, Topa A et al. (2012) Autonomic nervous system involvement in a new CMT2B family. J Peripher Nerv Syst 17 (3):361-364.
159. Fusco C, Ucchino V, Barbon G, Bonini E, Mostacciuolo ML, Frattini D et al. (2011) The homozygous ganglioside-induced differentiation-associated protein 1 mutation c.373C > T causes a very early-onset neuropathy: case report and literature review. J Child Neurol 26 (1):49-57.
160. Kalaydjieva L, Hallmayer J, Chandler D, Savov A, Nikolova A, Angelicheva D et al. (1996) Gene mapping in Gypsies identifies a novel demyelinating neuropathy on chromosome 8q24. Nat Genet 14 (2):214-217.
161. Merlini L, Villanova M, Sabatelli P, Trogu A, Malandrini A, Yanakiev P et al. (1998) Hereditary motor and sensory neuropathy Lom type in an Italian Gypsy family. Neuromuscul Disord 8 (3-4):182-185.

162. Rinaldi C, Grunseich C, Sevrioukova IF, Schindler A, Horkayne-Szakaly I, Lamperti C et al. (2012) Cowchock syndrome is associated with a mutation in apoptosis-inducing factor. Am J Hum Genet 91 (6):1095-1102.
163. Parman Y, Battaloglu E, Baris I, Bilir B, Poyraz M, Bissar-Tadmouri N et al. (2004) Clinicopathological and genetic study of early-onset demyelinating neuropathy. Brain 127 (Pt 11):2540-2550.
164. Sabatelli M, Mignogna T, Lippi G, Servidei S, Manfredi G, Ricci E et al. (1994) Autosomal recessive hypermyelinating neuropathy. Acta Neuropathol 87 (4):337-342.
165. Chance PF, Matsunami N, Lensch W, Smith B, Bird TD (1992) Analysis of the DNA duplication 17p11.2 in Charcot-Marie-Tooth neuropathy type 1 pedigrees: additional evidence for a third autosomal CMT1 locus. Neurology 42 (10):2037-2041.
166. Nicolaou P, Cianchetti C, Minaidou A, Marrosu G, Zamba-Papanicolaou E, Middleton L et al. (2013) A novel LRSAM1 mutation is associated with autosomal dominant axonal Charcot-Marie-Tooth disease. Eur J Hum Genet 21 (2):190-194.
167. Sanmaneechai O, Feely S, Scherer SS, Herrmann DN, Burns J, Muntoni F et al. (2015) Genotype-phenotype characteristics and baseline natural history of heritable neuropathies caused by mutations in the MPZ gene. Brain.
168. Chung KW, Kim SB, Park KD, Choi KG, Lee JH, Eun HW et al. (2006) Early onset severe and late-onset mild Charcot-Marie-Tooth disease with mitofusin 2 (MFN2) mutations. Brain 129 (Pt 8):2103-2118.
