# Supplementary material for: Underestimated associated features in CMT neuropathies: clinical indicators for the causative gene?
Source: Brain Behav. 2016 Mar 4;6(4):e00451. doi: 10.1002/brb3.451 (PMC4782242; doi:10.1002/brb3.451)
Supplement: Supplementary file 2 — Appendix S2. Phenotypic comparison of mutations found in our cohort with the literature. [file BRB3-6-e00451-s002.pdf]

Appendix S2. Phenotypic comparison of mutations found in our cohort with the literature.

	MPZ G163R		MPZ R98H		MPZ D224Y		LITAF V144M		GJB1 R183S		NEFL Q332P		INF2 L77R		MFN2 R280H		MFN2 R94Q	
	CS	Lit	CS	Lit	CS	Lit	CS	Lit	CS	Lit	CS	Lit	CS	Lit	CS	Lit	CS	Lit
CMT type	1	1 [1,2]	1, 2	1 [3]	1	1* [4]	1	1 [5]	1(M)	2(F) [6]	1	2 [7]	DI	1 [8]	2	2 [9,10]	2	2 [9,11]
Age of onset (y)	39, childhood	1st-7th decade [1,2]	20-25, 62	early-onset -60 [3]	40	AAE 31,78 [4]	29,49	10-57 [5]	childhood	na	26	2nd-3rd decade [7]	AAE 12	4 [8]	teens	11-35 [9,10],	5	2-17 [9,11]
Hypertrophic nerve roots	+	-	+	-	-	-	-	-	-	na	-	-	-	-	-	-	-	-
CSF protein elevation	+	-	+	[12]	-	[4]	+	-	-	na	-	-	-	-	-	-	-	-
Cataracts bilateral	+	-	+	-	-	-	-	-	-	na	-	-	-	-	-	-	-	-
CTS	+	[1]	-	-	-	-	+	-	-	na	-	-	-	-	-	-	-	-
Pain	-	-	-	[12]	-	-	+	[5]	+	na	-	-	-	-	+	[10]	-	-
Paresthesias	-	[1,2]	+	-	-	-	+	[5]	+	na	+	-	-	-	+	-	-	-
Tremor	+	-	-	-	-	[4]	+	-	+	na	-	-	-	-	-	[10]	+	[9]
Respiratory insufficiency	-	-	+	-	-	-	-	-	-	na	-	-	-	-	-	-	-	-
Scoliosis	+	-	-	-	-	-	+	-	-	na	-	-	-	-	-	-	-	-
Hip dysplasia	-	-	-	[13]	-	-	-	-	-	-	-	-	-	-	-	-	-	-
Fasciculations	-	-	+	-	-	-	+	-	-	na	-	[7]	-	-	-	-	-	-
FSGS	-	-	-	-	-	-	-	-	-	na	-	-	+	[8]	-	-	-	-
Pupillary abnormalities	+	-	-	-	-	-	-	-	-	na	-	-	-	-	-	-	-	[14]
RLS	-	-	-	-	-	-	+	-	-	na	-	-	-	-	+	-	-	-
BrainMRI abnormalities	-	-	+	-	-	-	-	-	-	na	-	-	-	[8]		[10]	-	-
Claw hands	-	-	+	-	-	-	-	-	-	na	-	-	-	-	-	-	-	-
Asymmetrical weakness	-	-	-	[12]	-	-	-	-	-	na	-	-	-	-	-	-	-	-
Autonomous	-	-	-	-	+	-	+	-	-	na	-	-	-	-	-	-	+	-
Severe slow NCV	-	-	-	-	-	-	-	-	-	na	-	-	-	-	-	-	-	-
UL predominant	-	-	-	-	-	-	+	-	-	na	-	-	-	-	-	-	-	-
Facial weakness	-	-	-	-	-	-	-	-	-	na	-	[7]	-	-	-	-	-	-
Deafness	-	-	-	-	-	-	-	-	-	na	-	-	-	[8]	-	[10]	-	-
Hyperkeratosis	-	-	-	-	-	-	-	-	-	na	-	[7]	-	-	-	-	-	-
Syndactyly	-	-	-	-	-	-	-	-	-	na	-	[7]	-	-	-	-	-	-
Cold induced hand cramps	-	-	-	-	-	-	-	-	-	na	-	-	-	-	+	-	-	-
Bulbar	-	-	-	-	-	-	-	-	-	na	-	-	-	-	-	[10]	-	-
Upper motor neuron	-	-	-	-	-	-	-	-	-	na	-	-	-	-	-	[10]	-	-
Migraine	-	-	-	-	-	-	-	-	-	na	-	-	-	-	-	[10]	-	-
Cognitive impairment	-	-	-	-	-	-	+	-	-	na	-	-	-	[8]	-	-	-	-
MS as additional diagnosis	-	-	-	-	-	[4]*	+	-	-	na	-	-	-	-	-	-	-	-

CS, current study; Lit, literature; CMT, Charcot-Marie-Tooth neuropathy; *homozygous patient(s); M, male; F, female; na, not available; DI, dominant intermediate; AAE, age at examination; CSF, cerebrospinal fluid; CTS, carpal tunnel syndrome; FSGS, focal segmental glomerular sclerosis; RLS, restless legs syndrome; MRI, magnetic resonance imaging; NCV, nerve conduction velocity; UL, upper limb; MS, multiple sclerosis. For abbreviations of genes see text.

References:

1. Street VA, Meekins G, Lipe HP, Seltzer WK, Carter GT, Kraft GH et al. (2002) Charcot-Marie-Tooth neuropathy: clinical phenotypes of four novel mutations in the MPZ and Cx 32 genes. Neuromuscul Disord 12 (7-8):643-650.
2. Eggers SD, Keswani SC, Melli G, Cornblath DR (2004) Clinical and genetic description of a family with Charcot-Marie-Tooth disease type 1B from a transmembrane MPZ mutation. Muscle Nerve 29 (6):867-869.
3. Gabreels-Festen AA, Hoogendijk JE, Meijerink PH, Gabreels FJ, Bolhuis PA, van Beersum S et al. (1996) Two divergent types of nerve pathology in patients with different P0 mutations in Charcot-Marie-Tooth disease. Neurology 47 (3):761-765.
4. Fabrizi GM, Pellegrini M, Angiari C, Cavallaro T, Morini A, Taioli F et al. (2006) Gene dosage sensitivity of a novel mutation in the intracellular domain of P0 associated with Charcot-Marie-Tooth disease type 1B. Neuromuscul Disord 16 (3):183-187.
5. Gerding WM, Koetting J, Epplen JT, Neusch C (2009) Hereditary motor and sensory neuropathy caused by a novel mutation in LITAF. Neuromuscul Disord 19 (10):701-703.
6. Bort S, Nelis E, Timmerman V, Sevilla T, Cruz-Martinez A, Martinez F et al. (1997) Mutational analysis of the MPZ, PMP22 and Cx32 genes in patients of Spanish ancestry with Charcot-Marie-Tooth disease and hereditary neuropathy with liability to pressure palsies. Hum Genet 99 (6):746-754.
7. Mersiyanova IV, Perepelov AV, Polyakov AV, Sitnikov VF, Dadali EL, Oparin RB et al. (2000) A new variant of Charcot-Marie-Tooth disease type 2 is probably the result of a mutation in the neurofilament-light gene. Am J Hum Genet 67 (1):37-46.
8. Mademan I, Deconinck T, Dinopoulos A, Voit T, Schara U, Devriendt K et al. (2013) De novo INF2 mutations expand the genetic spectrum of hereditary neuropathy with glomerulopathy. Neurology 81 (22):1953-1958.
9. Zuchner S, Mersiyanova IV, Muglia M, Bissar-Tadmouri N, Rochelle J, Dadali EL et al. (2004) Mutations in the mitochondrial GTPase mitofusin 2 cause Charcot-Marie-Tooth neuropathy type 2A. Nat Genet 36 (5):449-451.
10. Chung KW, Kim SB, Park KD, Choi KG, Lee JH, Eun HW et al. (2006) Early onset severe and late-onset mild Charcot-Marie-Tooth disease with mitofusin 2 (MFN2) mutations. Brain 129 (Pt 8):2103-2118.
11. Verhoeven K, Claeys KG, Zuchner S, Schroder JM, Weis J, Ceuterick C et al. (2006) MFN2 mutation distribution and genotype/phenotype correlation in Charcot-Marie-Tooth type 2. Brain 129 (Pt 8):2093-2102.
12. Watanabe M, Yamamoto N, Ohkoshi N, Nagata H, Kohno Y, Hayashi A et al. (2002) Corticosteroid- responsive asymmetric neuropathy with a myelin protein zero gene mutation. Neurology 59 (5):767-769.
13. Sanmaneechai O, Feely S, Scherer SS, Herrmann DN, Burns J, Muntoni F et al. (2015) Genotype-phenotype characteristics and baseline natural history of heritable neuropathies caused by mutations in the MPZ gene. Brain.
14. Houlden H, Reilly MM, Smith S (2009) Pupil abnormalities in 131 cases of genetically defined inherited peripheral neuropathy. Eye (Lond) 23 (4):966-974.
